# Supplementary figures and images for: The protective roles of eugenol on type 1 diabetes mellitus through NRF2-mediated oxidative stress pathway
Source: eLife. 2025 Jan 10;13:RP96600. doi: 10.7554/eLife.96600 (PMC11723580; doi:10.7554/eLife.96600)

Full unedited gel for Figure 2A. The red box shows the image used in the manuscript.

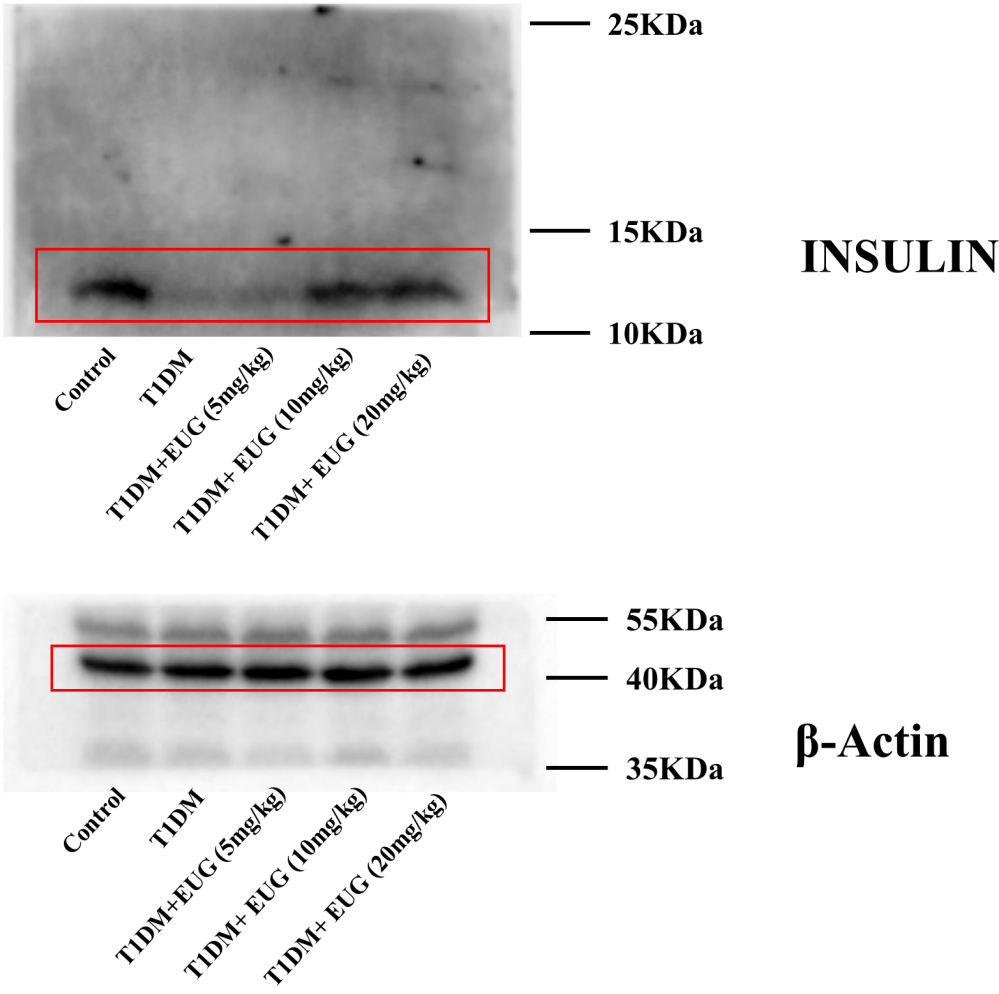

Supplement: Figure 2—source data 1. [file elife-96600-fig2-data1.pdf]

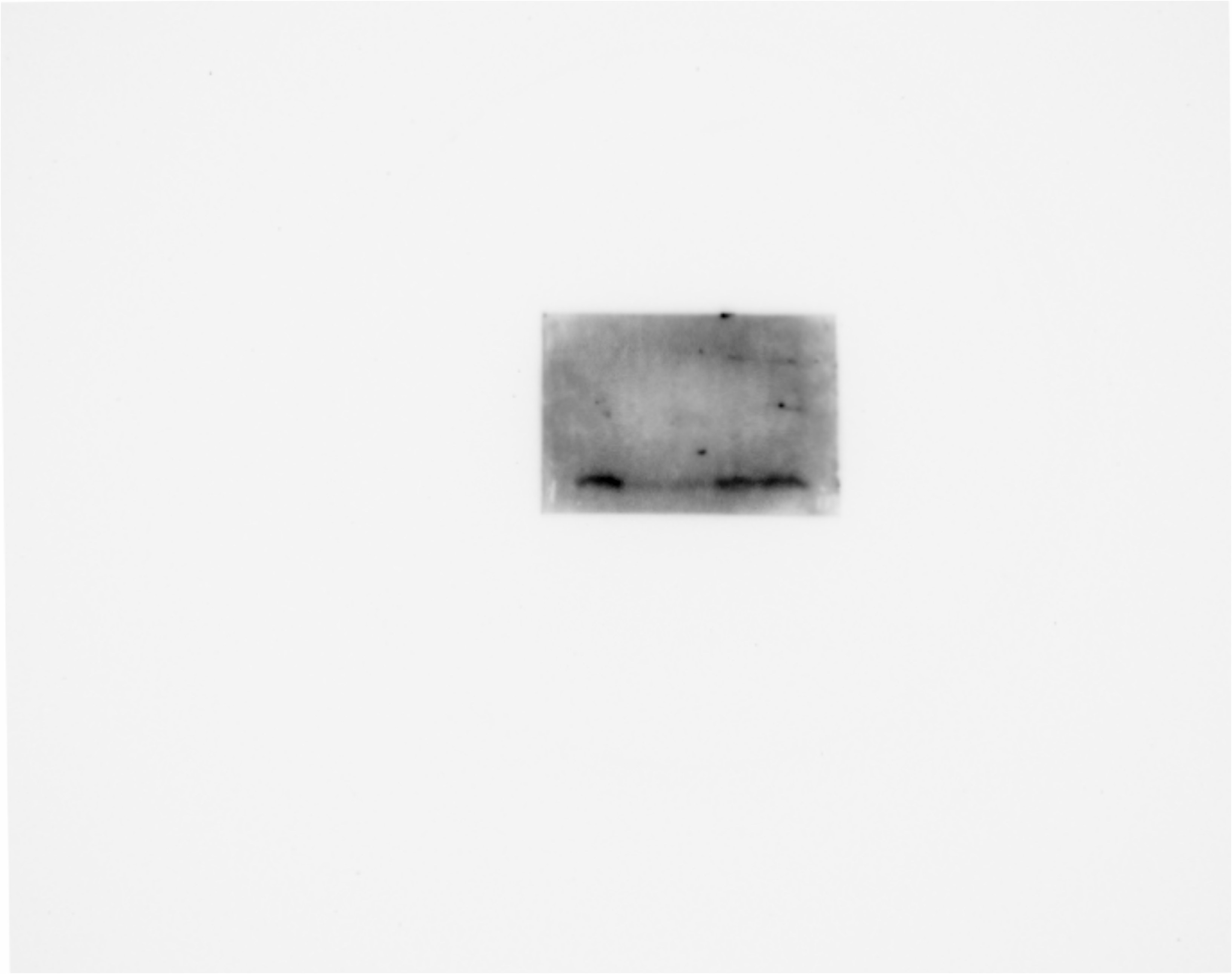

Supplement: Figure 2—source data 2. [file elife-96600-fig2-data2.zip › Raw unedited gels for Figure 2A/INSULIN.tif]

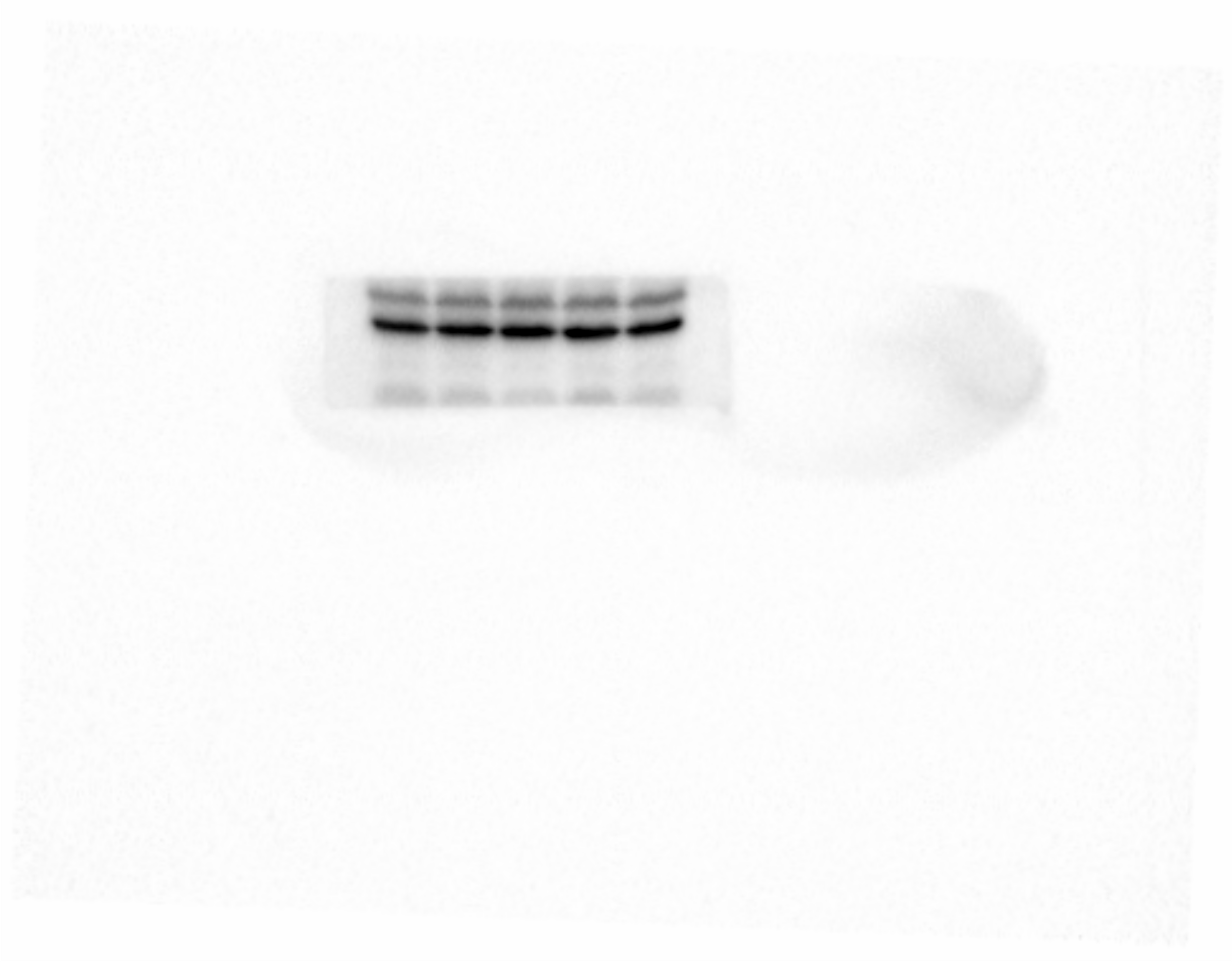

Supplement: Figure 2—source data 2. [file elife-96600-fig2-data2.zip › Raw unedited gels for Figure 2A/β-Actin.tif]

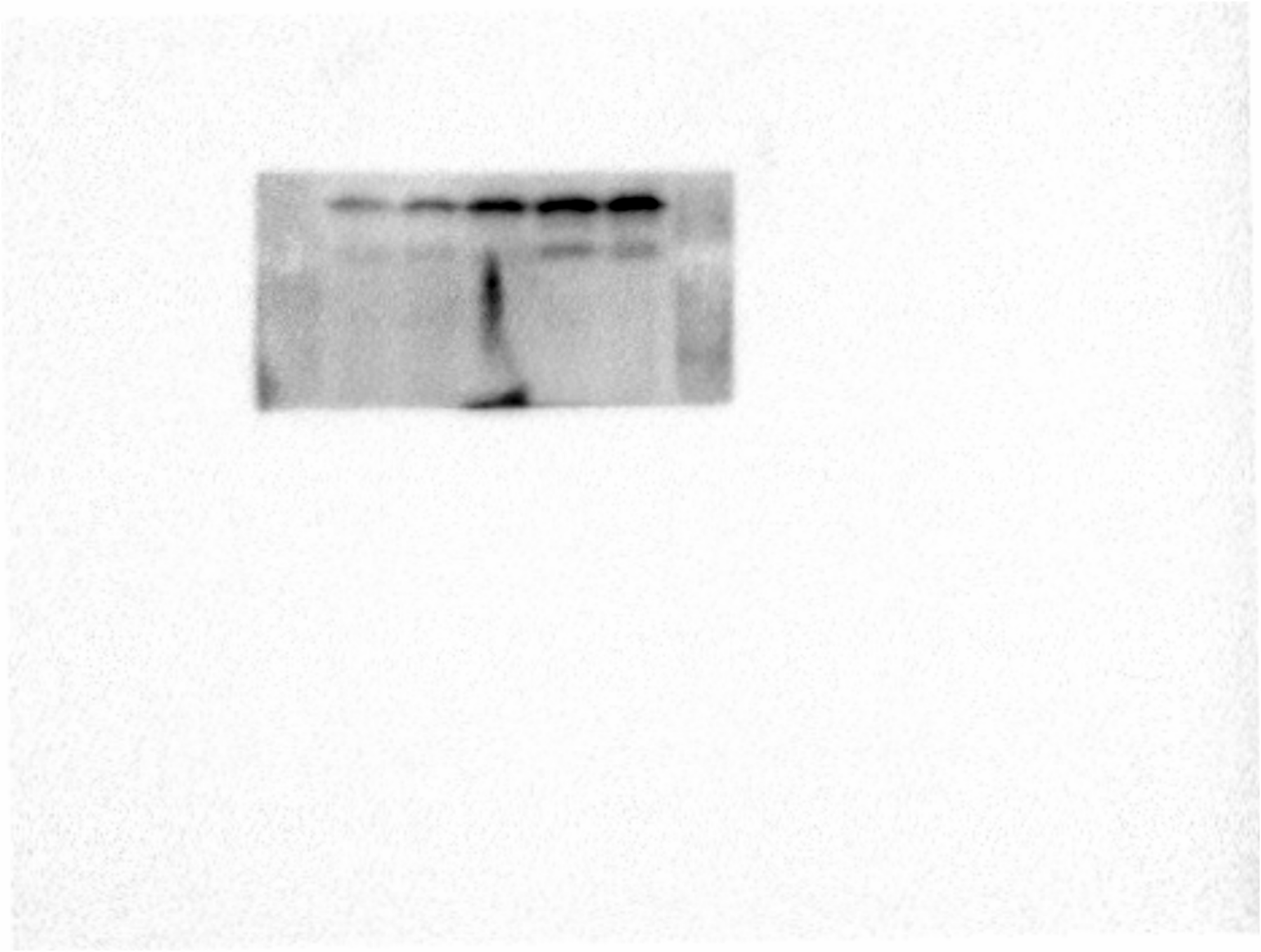

Supplement: Figure 3—source data 2. [file elife-96600-fig3-data2.zip › Raw unedited gels for Figure 3A-B/HMOX1.tif]

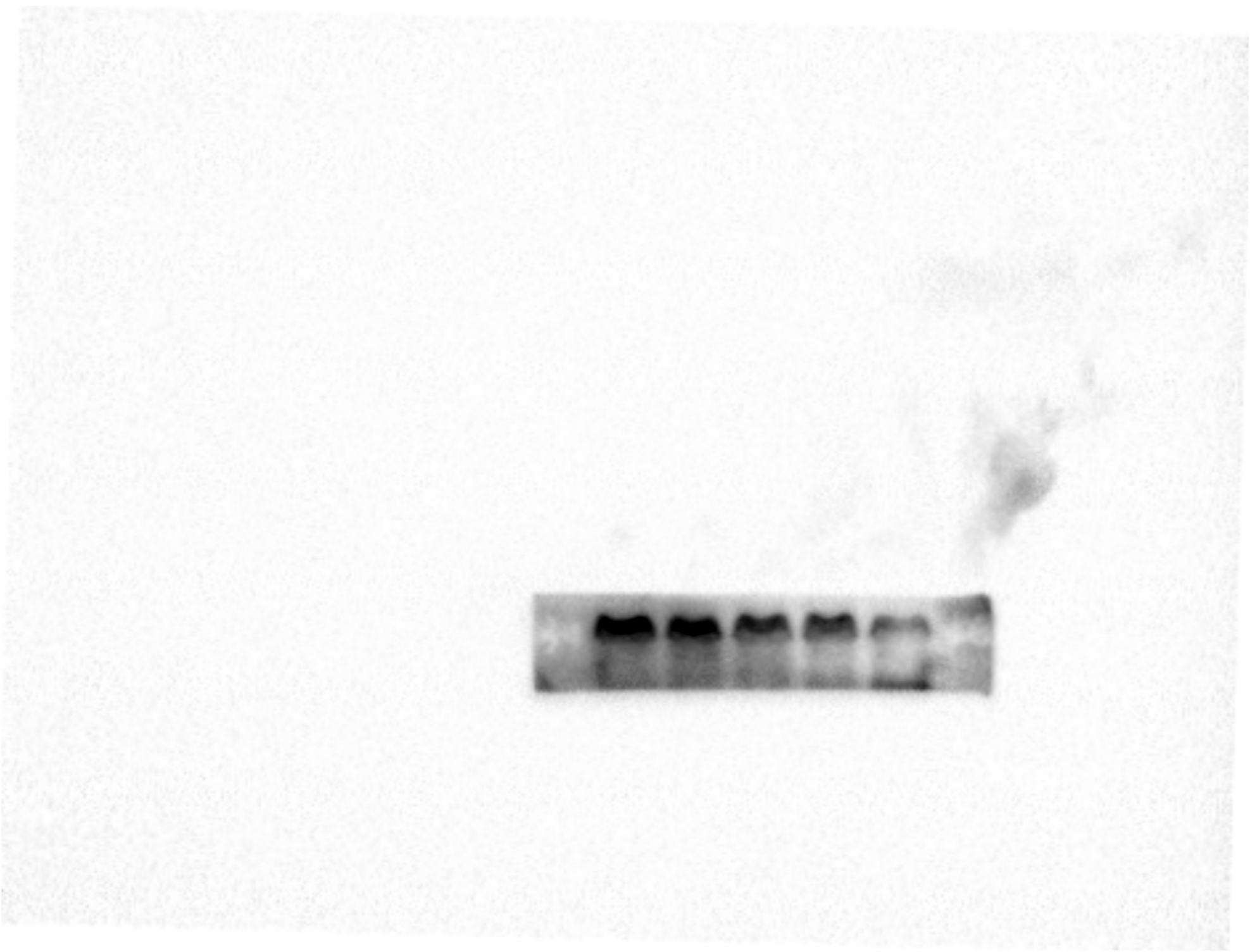

Supplement: Figure 3—source data 2. [file elife-96600-fig3-data2.zip › Raw unedited gels for Figure 3A-B/KEAP1.tif]

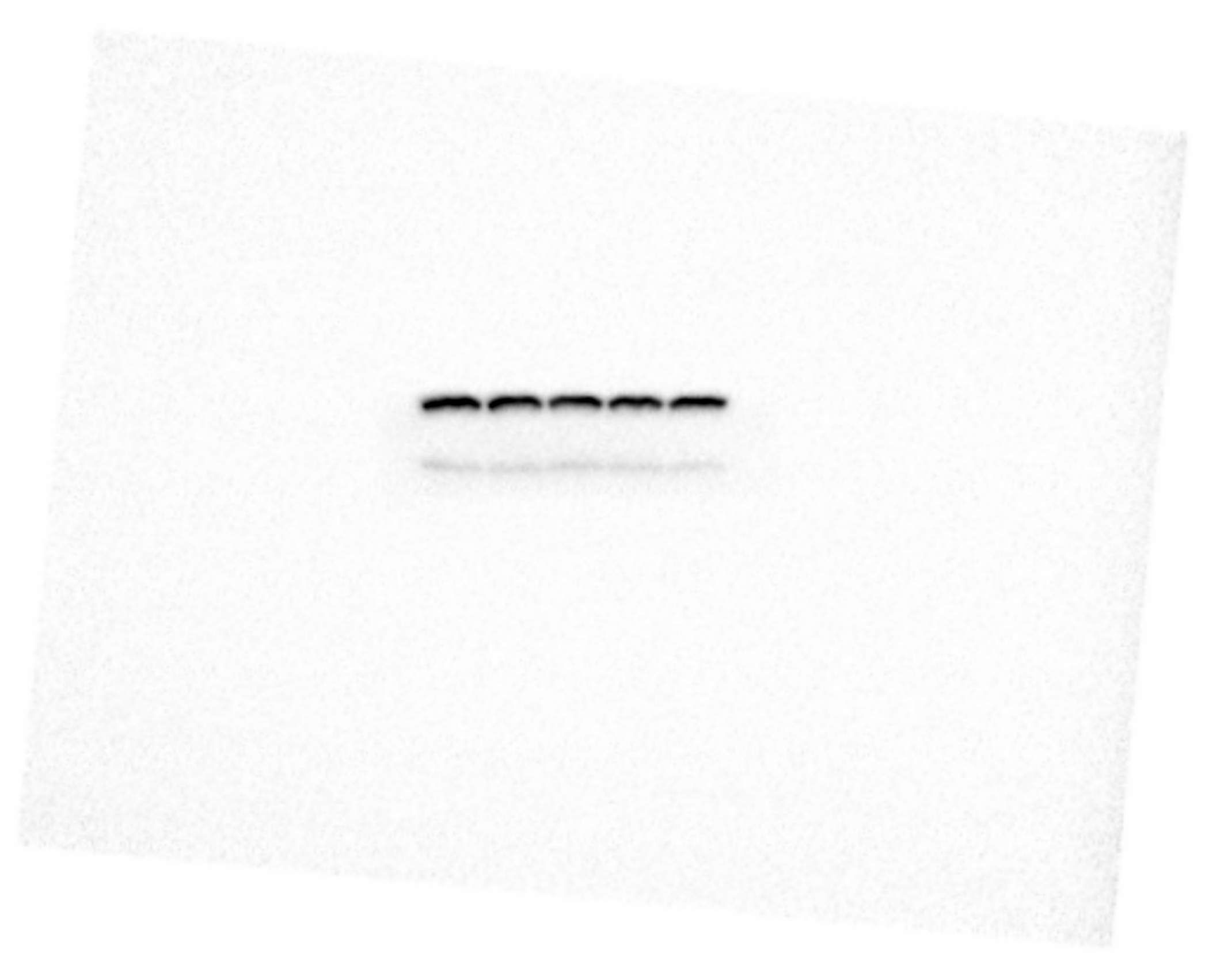

Supplement: Figure 3—source data 2. [file elife-96600-fig3-data2.zip › Raw unedited gels for Figure 3A-B/Lamin B.tif]

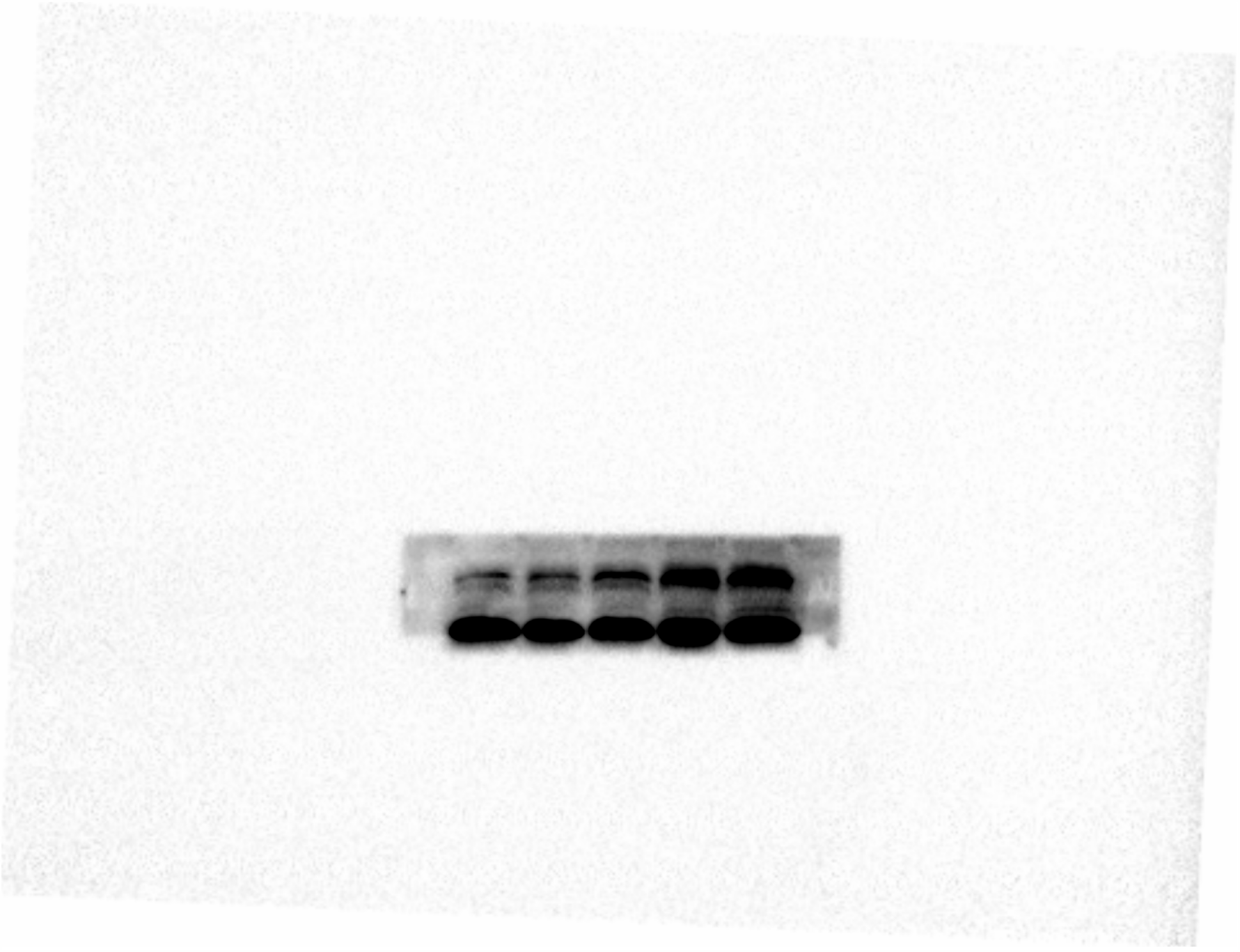

Supplement: Figure 3—source data 2. [file elife-96600-fig3-data2.zip › Raw unedited gels for Figure 3A-B/N-NRF2.tif]

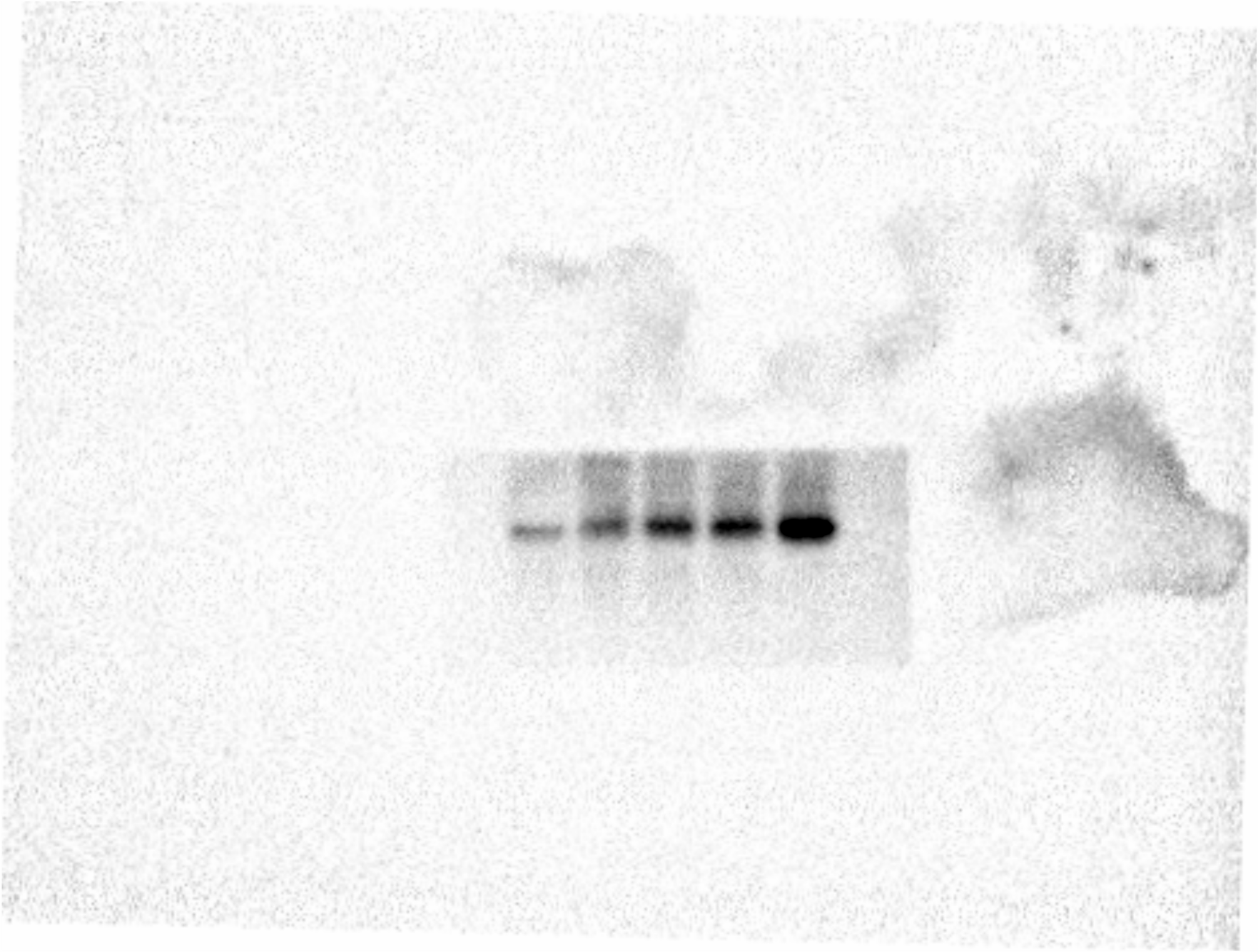

Supplement: Figure 3—source data 2. [file elife-96600-fig3-data2.zip › Raw unedited gels for Figure 3A-B/NQO1.tif]

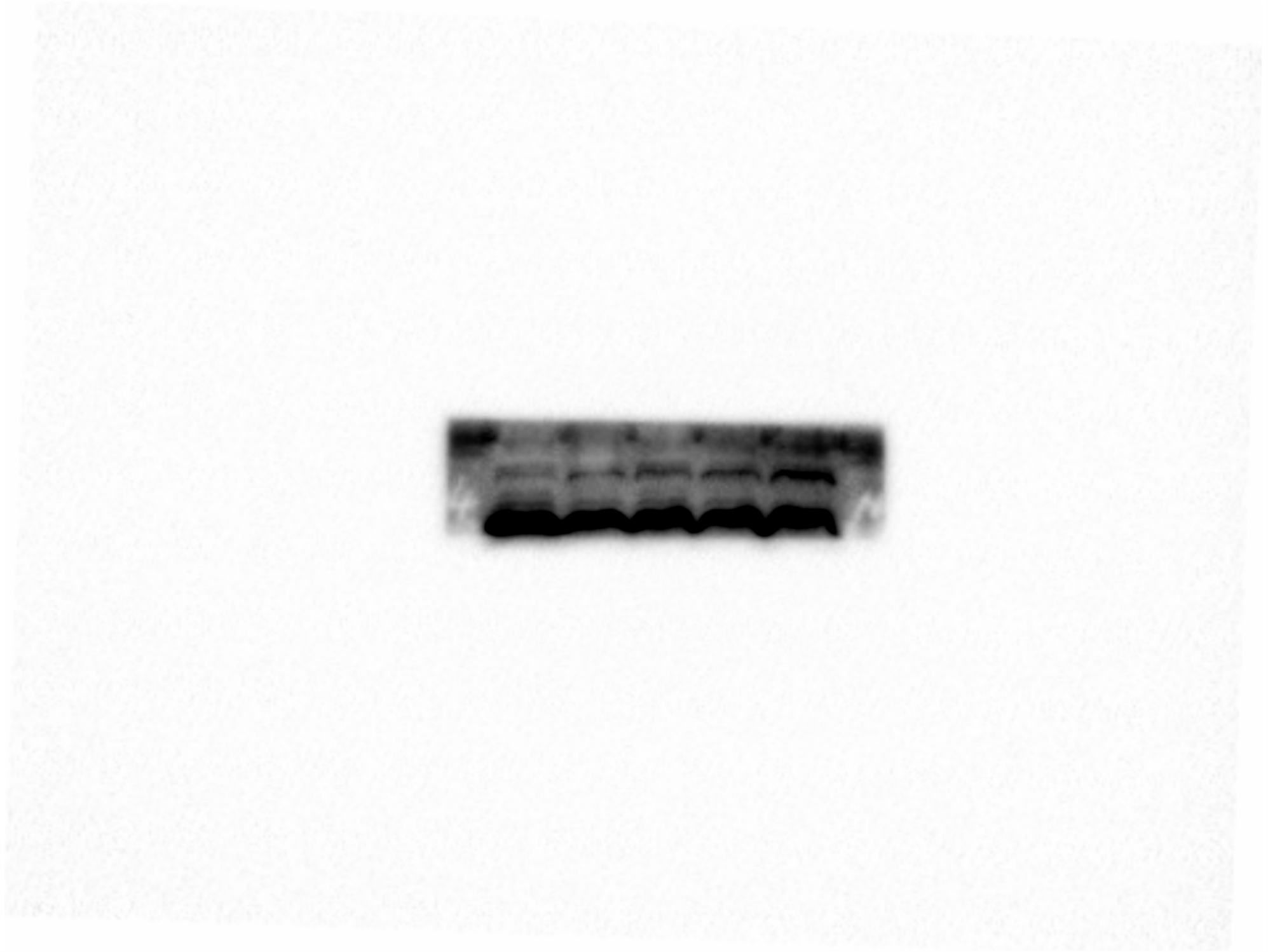

Supplement: Figure 3—source data 2. [file elife-96600-fig3-data2.zip › Raw unedited gels for Figure 3A-B/T-NFR2.tif]

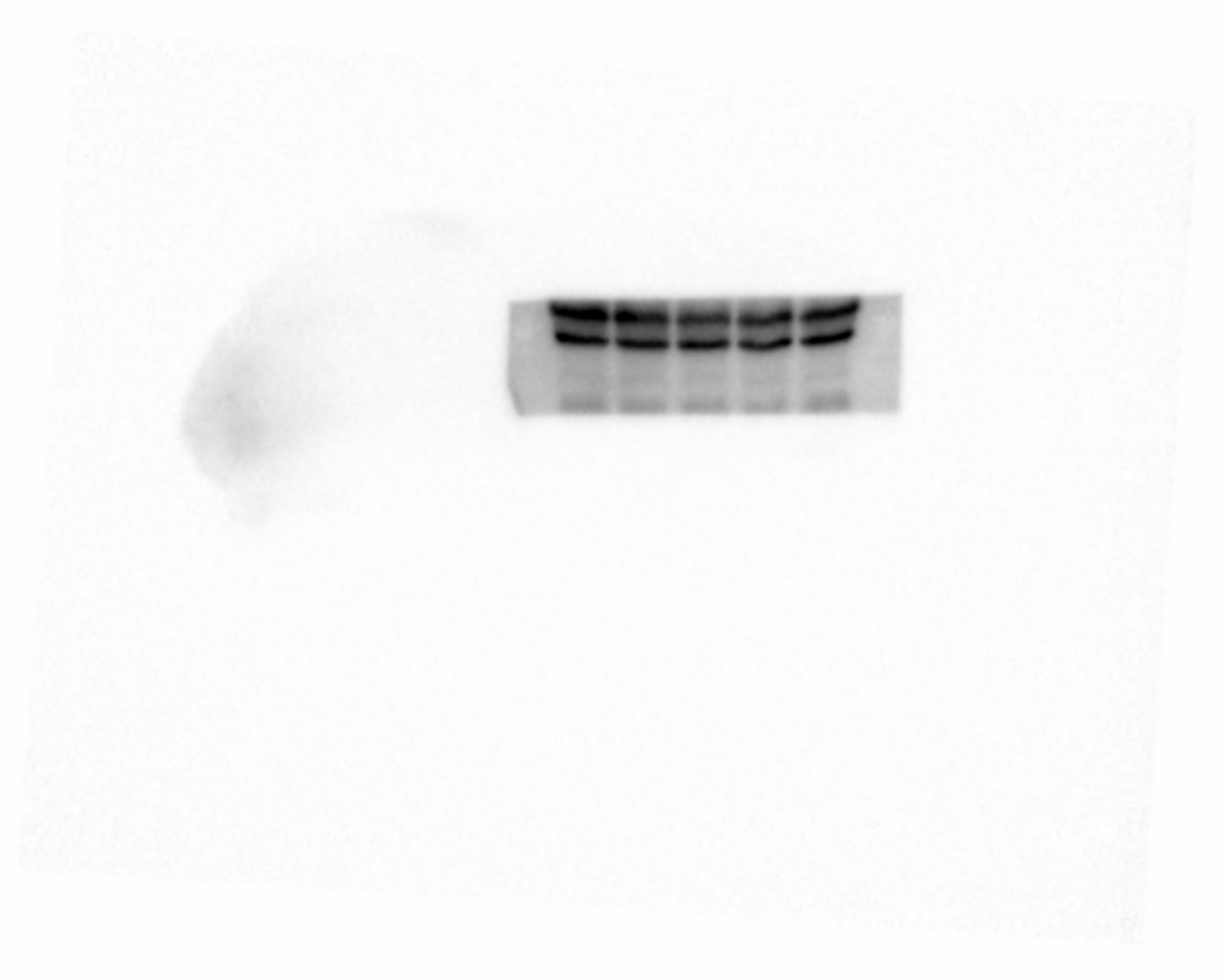

Supplement: Figure 3—source data 2. [file elife-96600-fig3-data2.zip › Raw unedited gels for Figure 3A-B/β-Actin-HMOX1.tif]

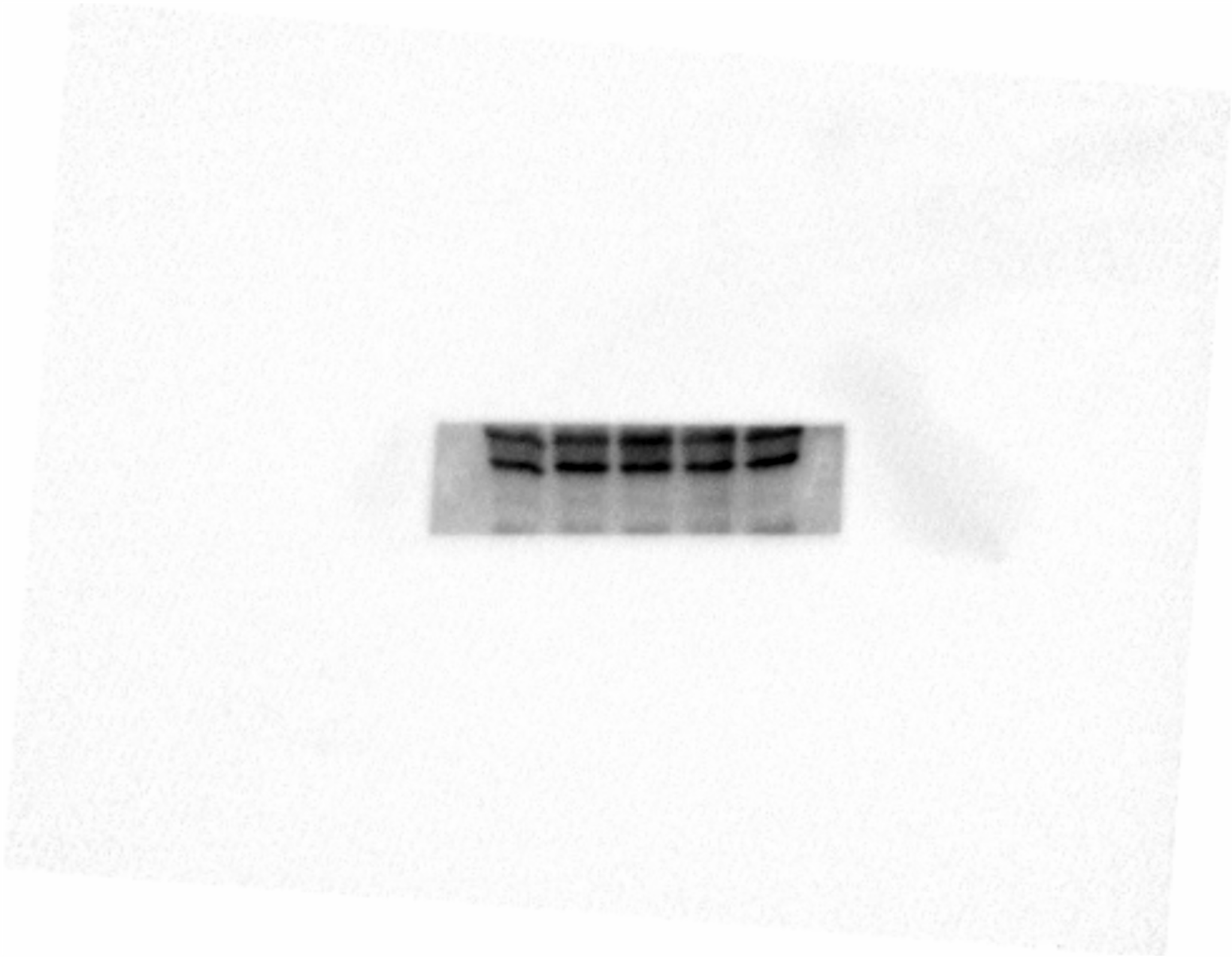

Supplement: Figure 3—source data 2. [file elife-96600-fig3-data2.zip › Raw unedited gels for Figure 3A-B/β-Actin-KEAP1.tif]

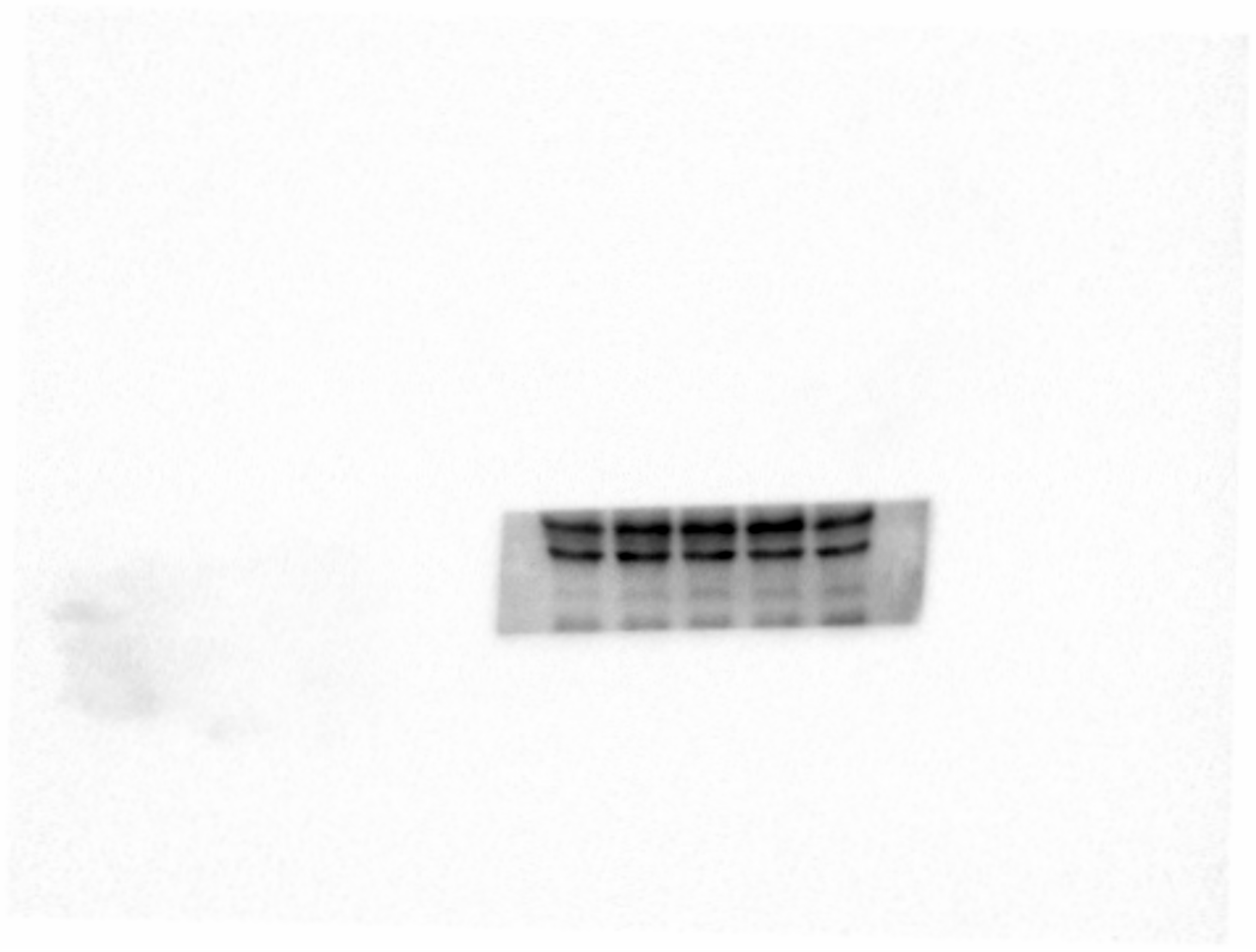

Supplement: Figure 3—source data 2. [file elife-96600-fig3-data2.zip › Raw unedited gels for Figure 3A-B/β-Actin-NQO1.tif]

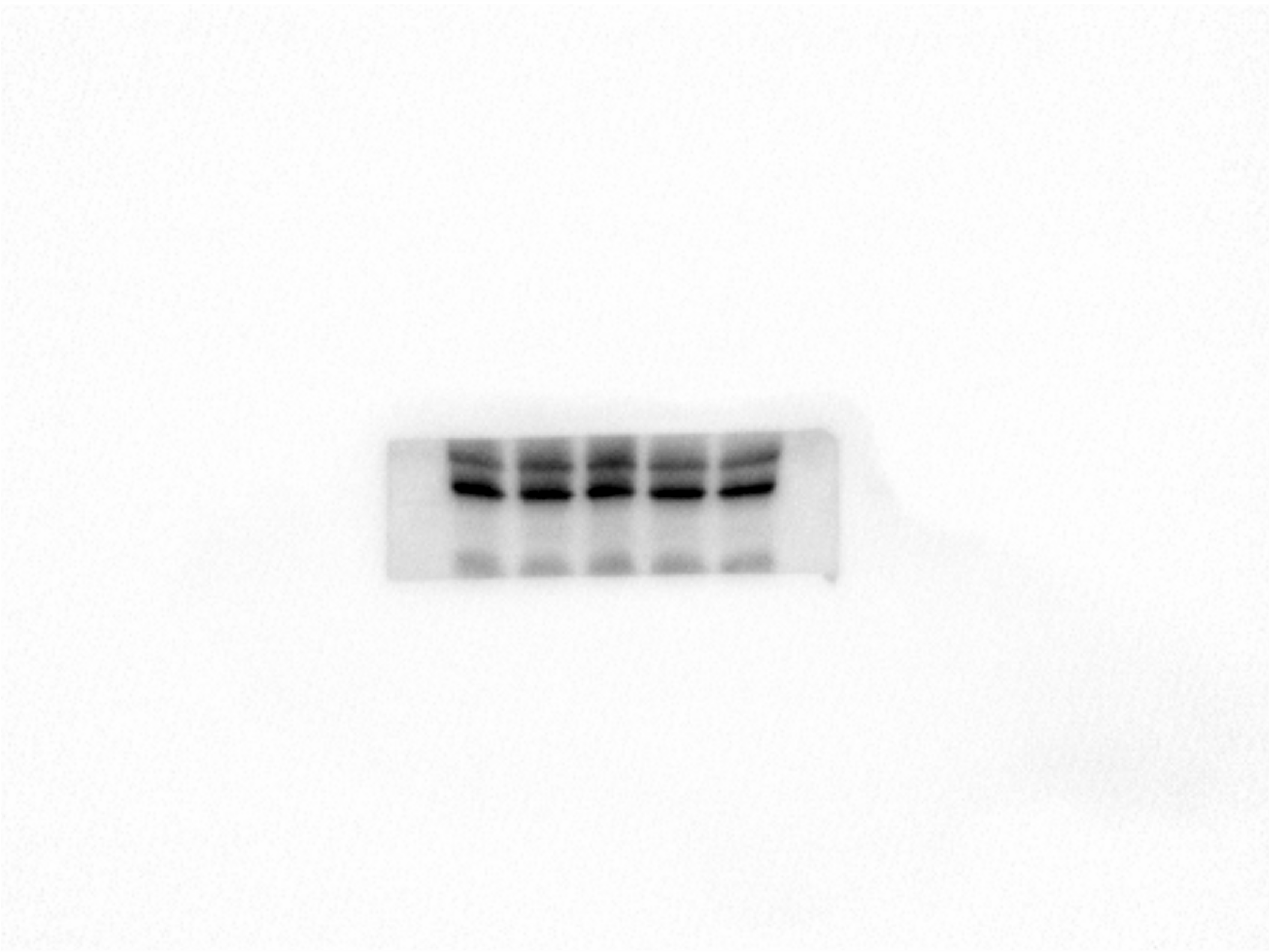

Supplement: Figure 3—source data 2. [file elife-96600-fig3-data2.zip › Raw unedited gels for Figure 3A-B/β-Actin.tif]

Full unedited gel for Figure 4A. The red box shows the image used in the manuscript.

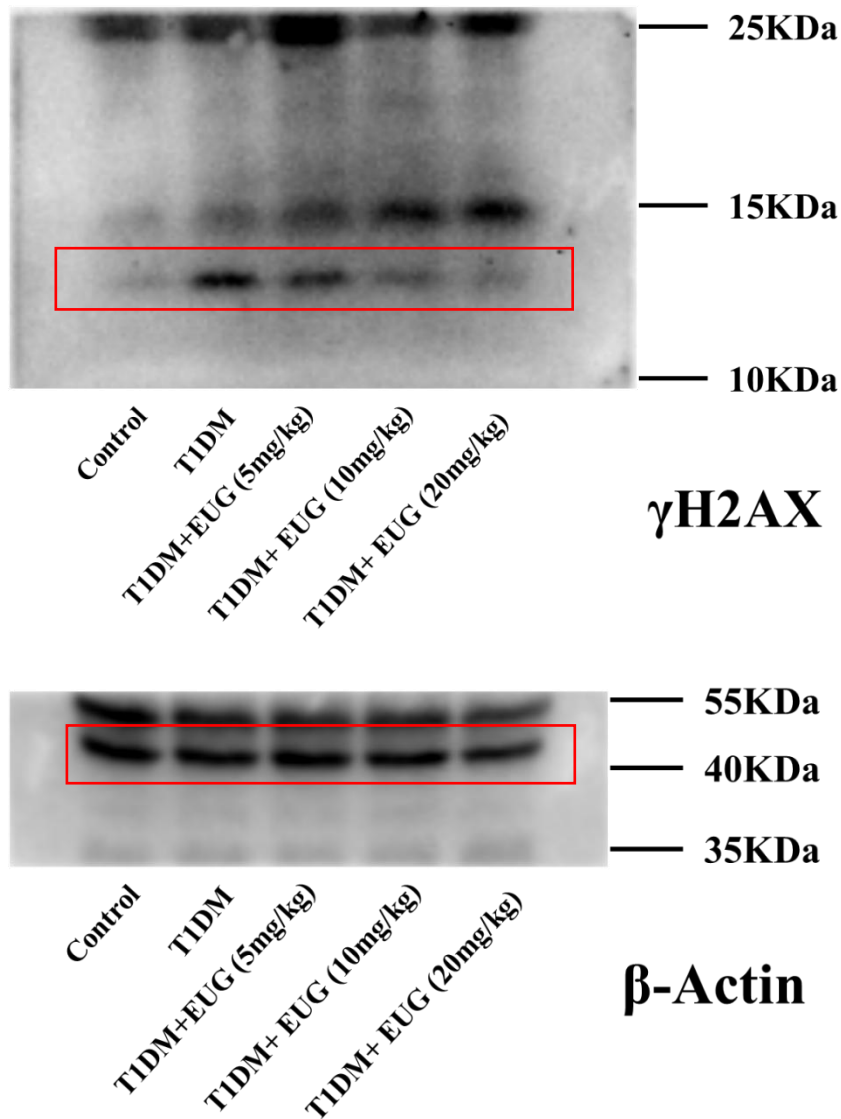

Supplement: Figure 4—source data 1. [file elife-96600-fig4-data1.pdf]

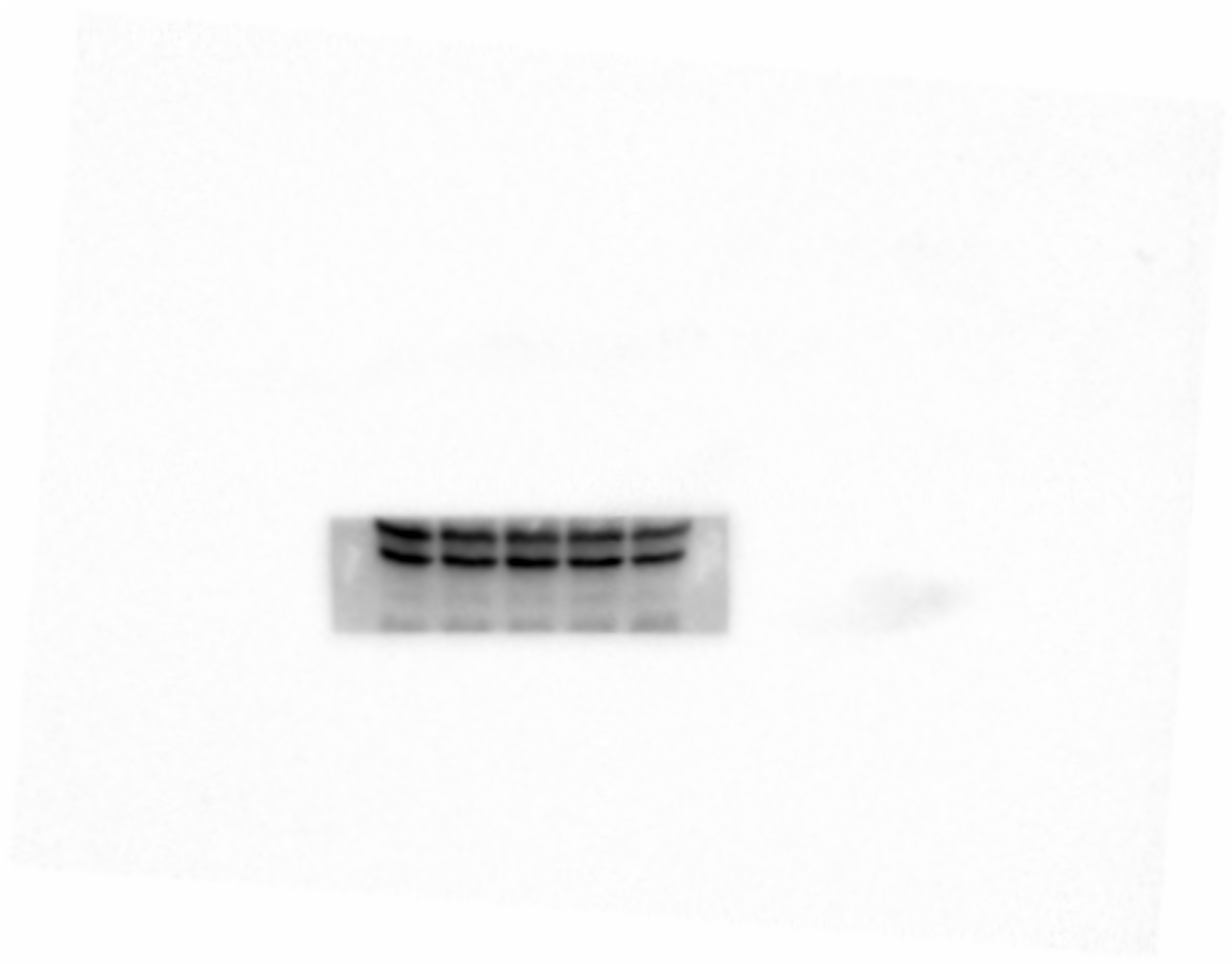

Supplement: Figure 4—source data 2. [file elife-96600-fig4-data2.zip › Raw unedited gels for Figure 4A/β-Actin.tif]

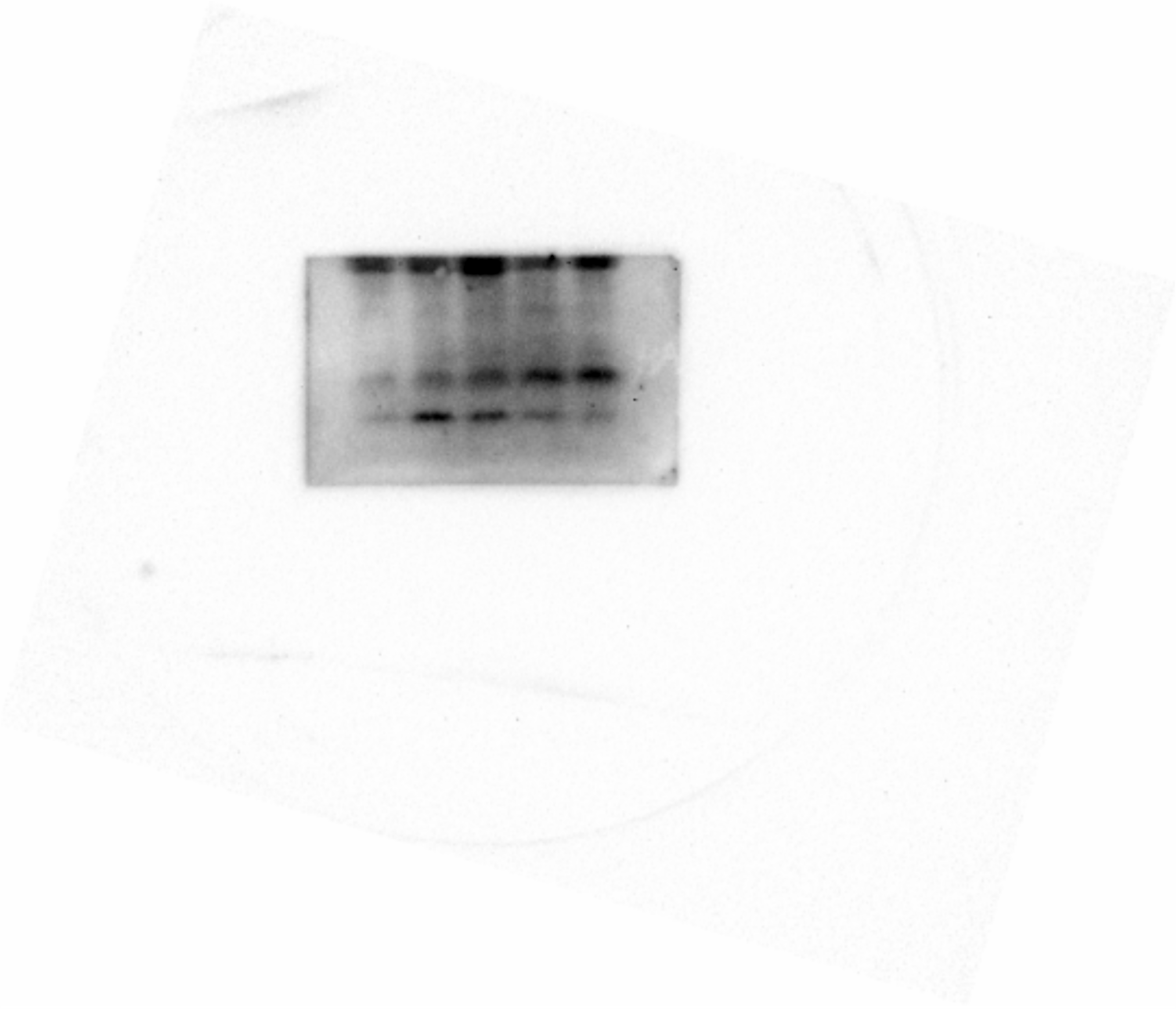

Supplement: Figure 4—source data 2. [file elife-96600-fig4-data2.zip › Raw unedited gels for Figure 4A/γH2AX.tif]

Full unedited gel for Figure 5A. The red box shows the image used in the manuscript.

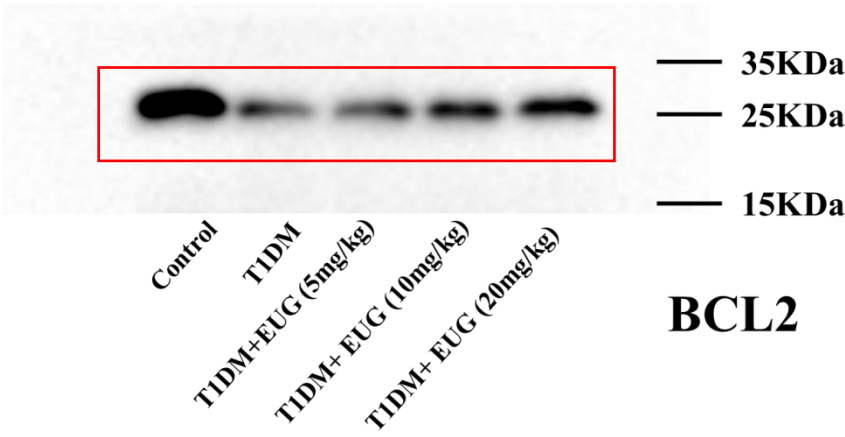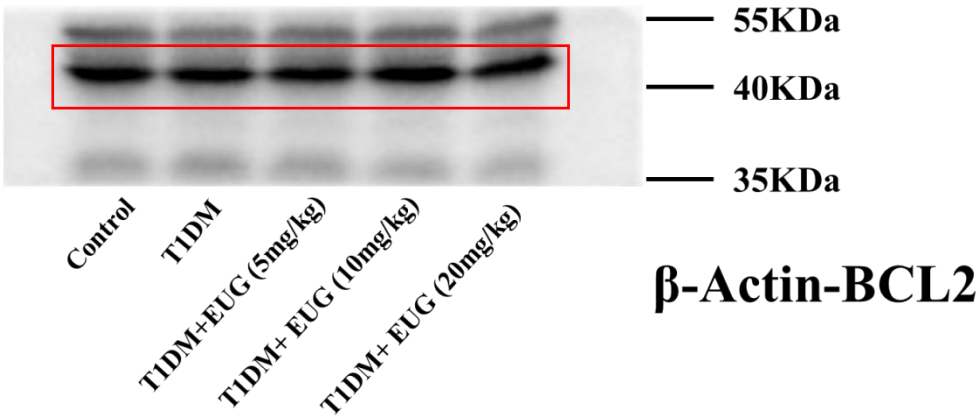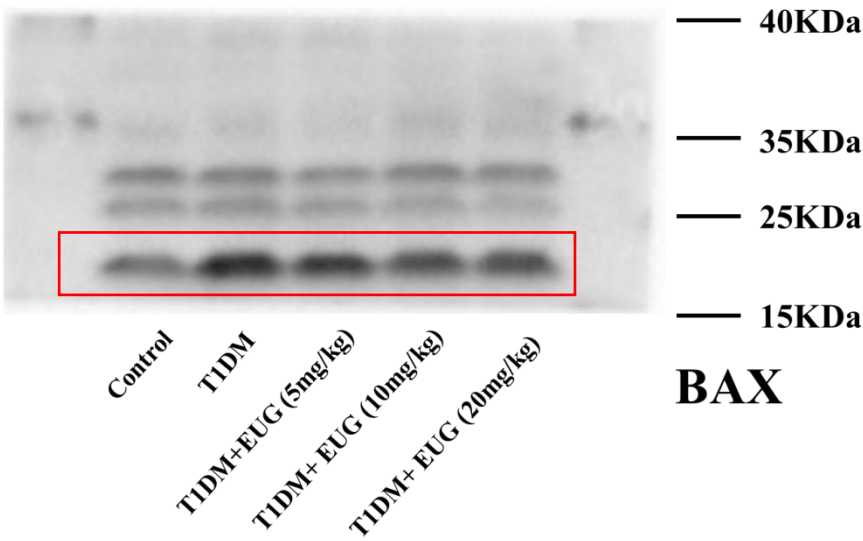

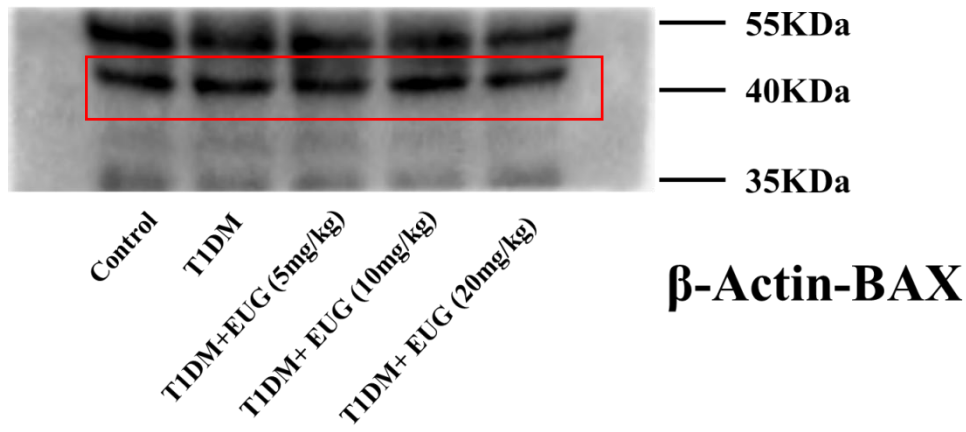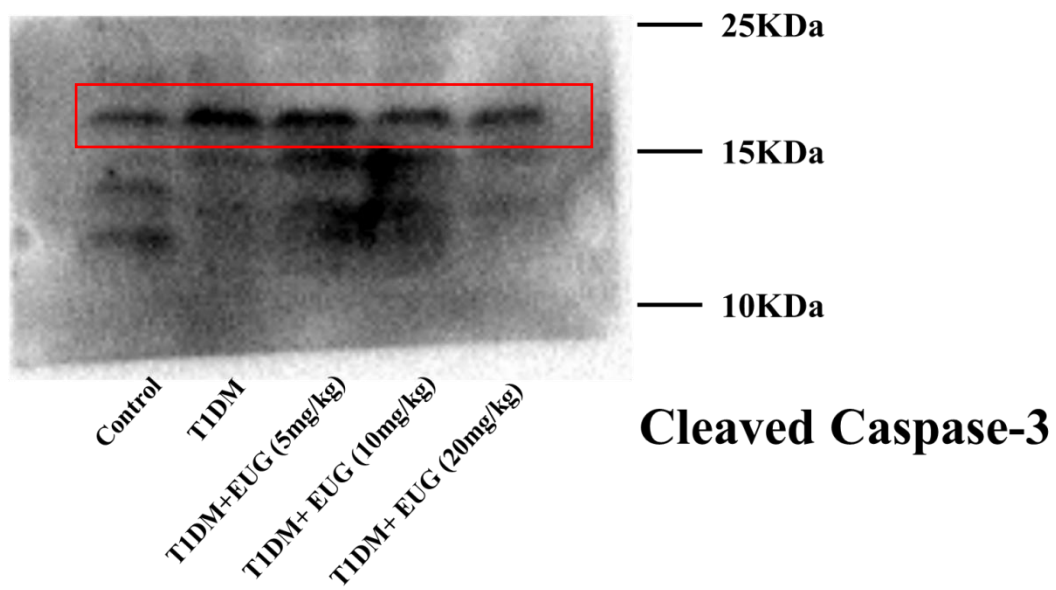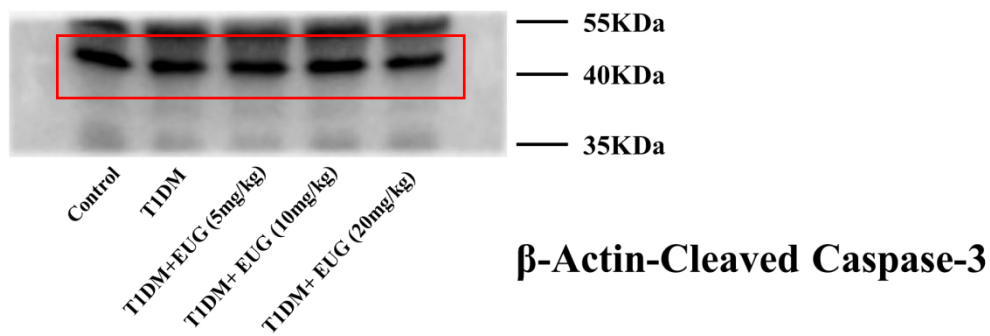

Supplement: Figure 5—source data 1. [file elife-96600-fig5-data1.pdf]

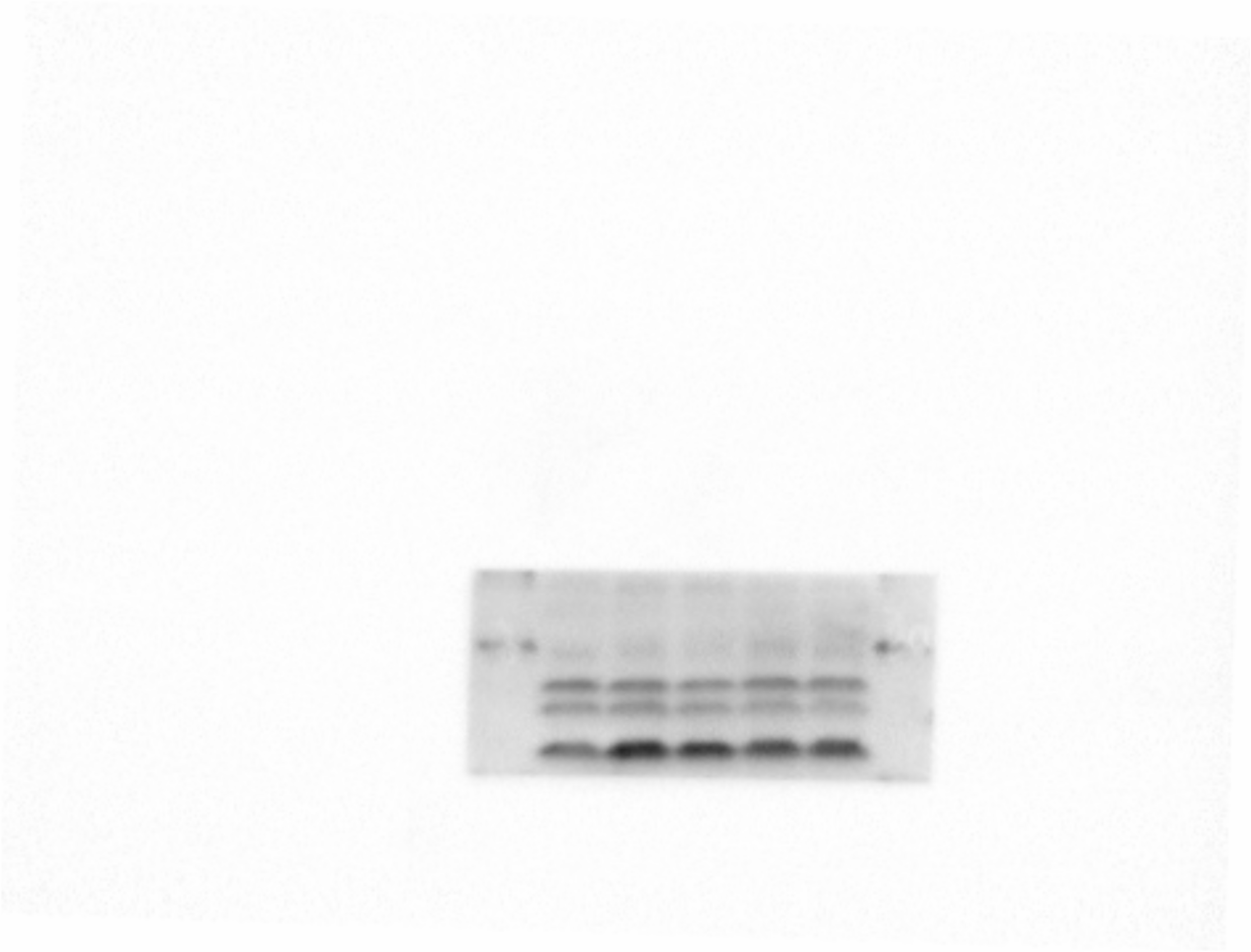

Supplement: Figure 5—source data 2. [file elife-96600-fig5-data2.zip › Raw unedited gels for Figure 5A/BAX.tif]

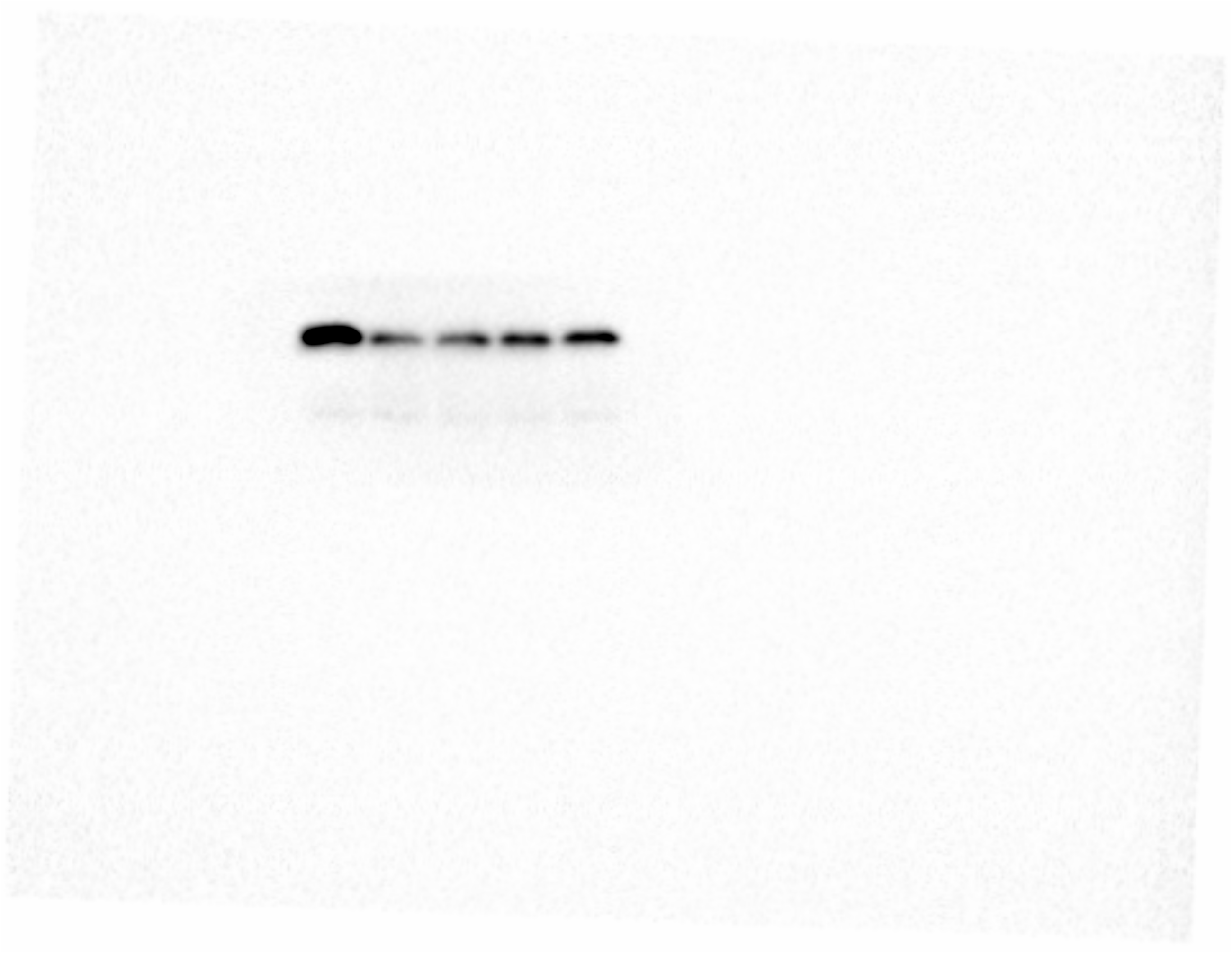

Supplement: Figure 5—source data 2. [file elife-96600-fig5-data2.zip › Raw unedited gels for Figure 5A/BCL2.tif]

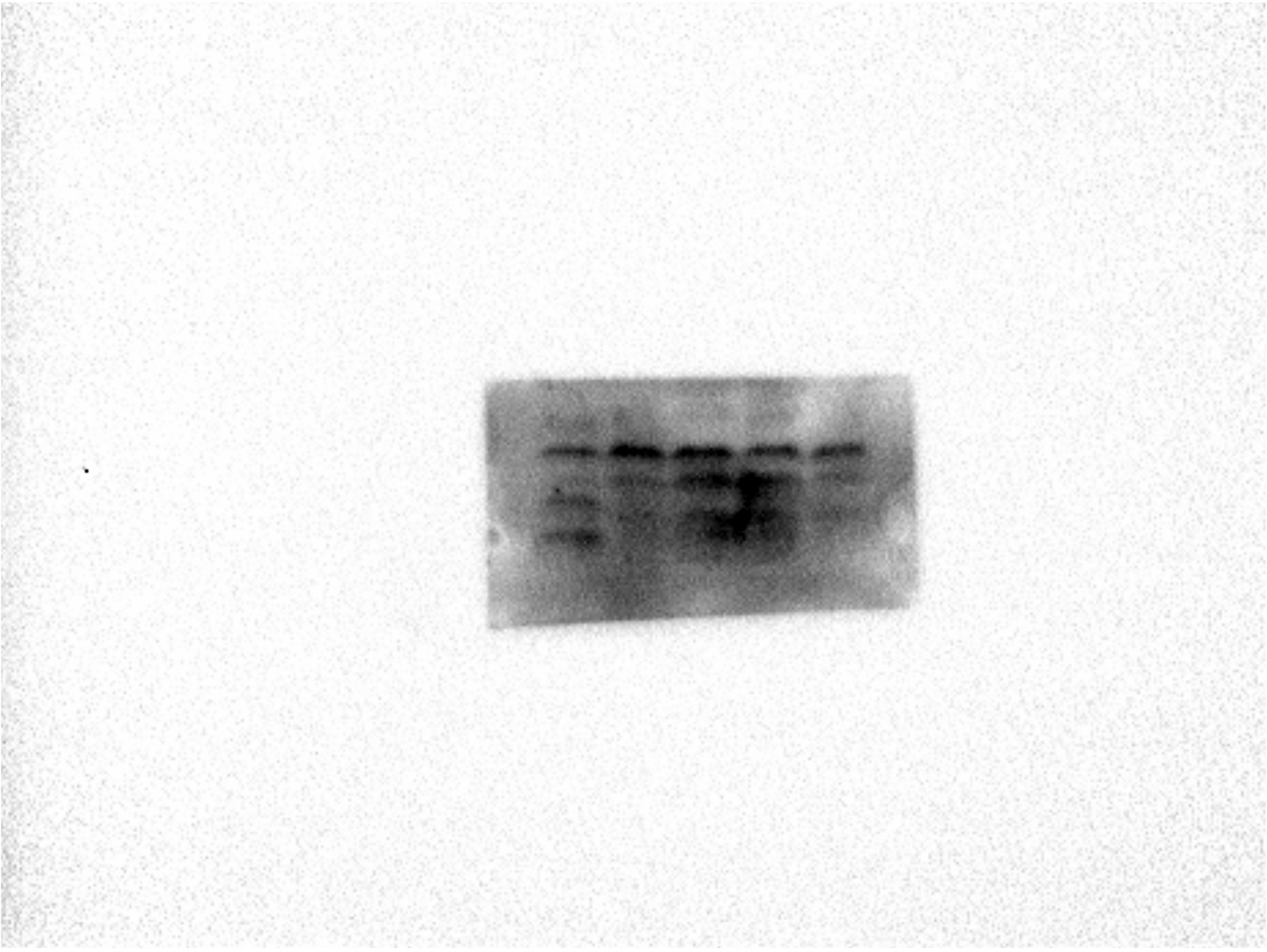

Supplement: Figure 5—source data 2. [file elife-96600-fig5-data2.zip › Raw unedited gels for Figure 5A/Cleaved Casepase 3.tif]

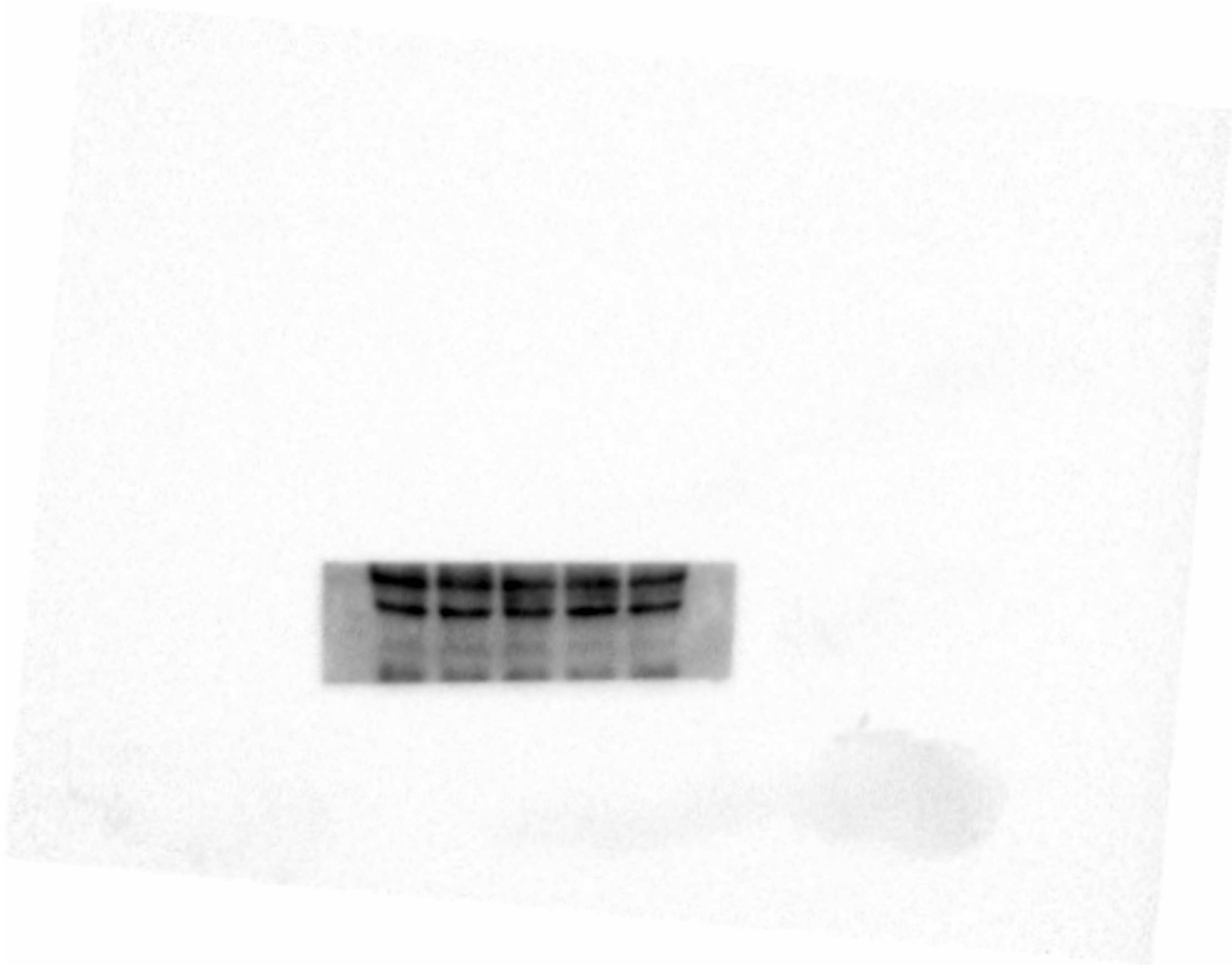

Supplement: Figure 5—source data 2. [file elife-96600-fig5-data2.zip › Raw unedited gels for Figure 5A/β-Actin-BAX.tif]

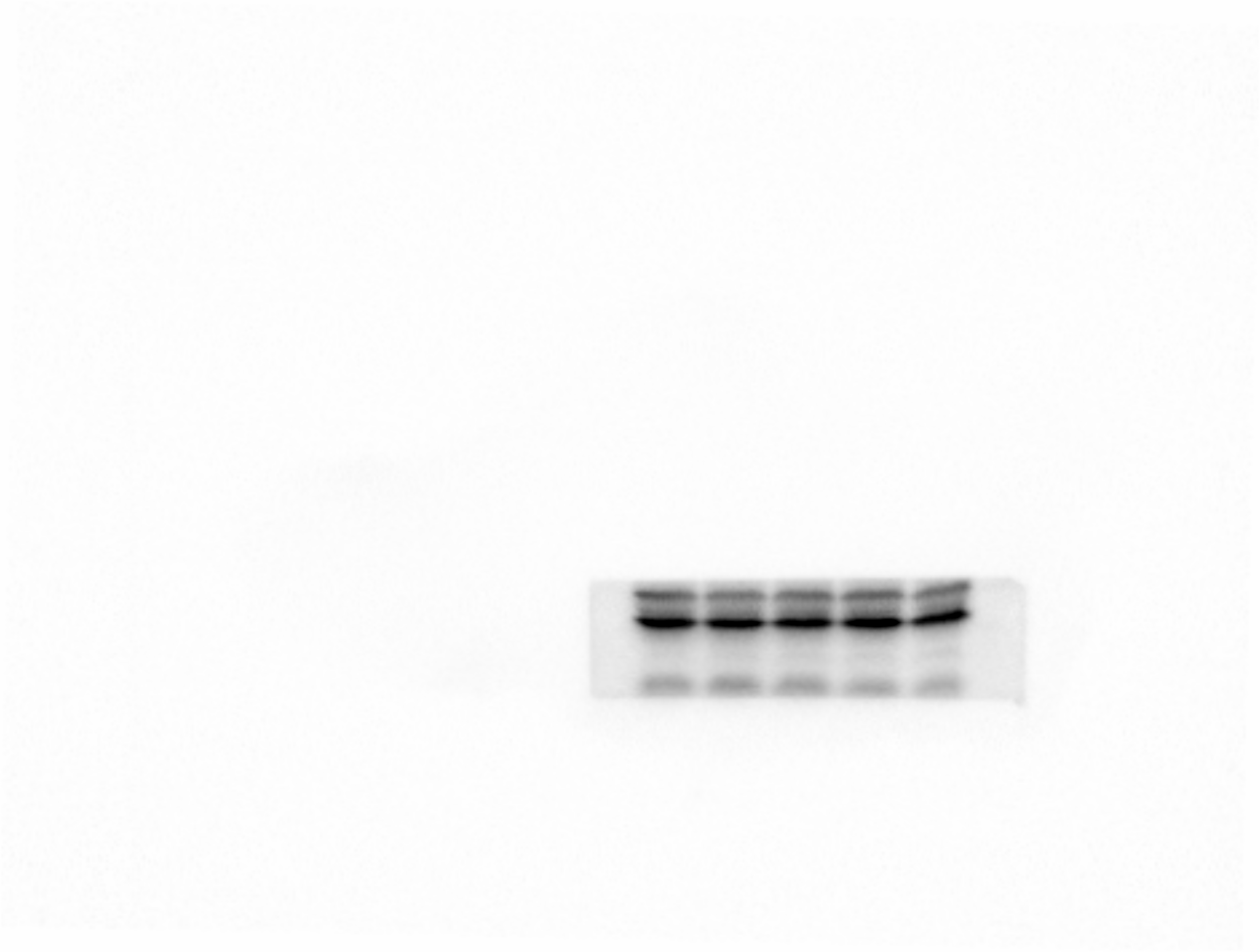

Supplement: Figure 5—source data 2. [file elife-96600-fig5-data2.zip › Raw unedited gels for Figure 5A/β-Actin-BCL2.tif]

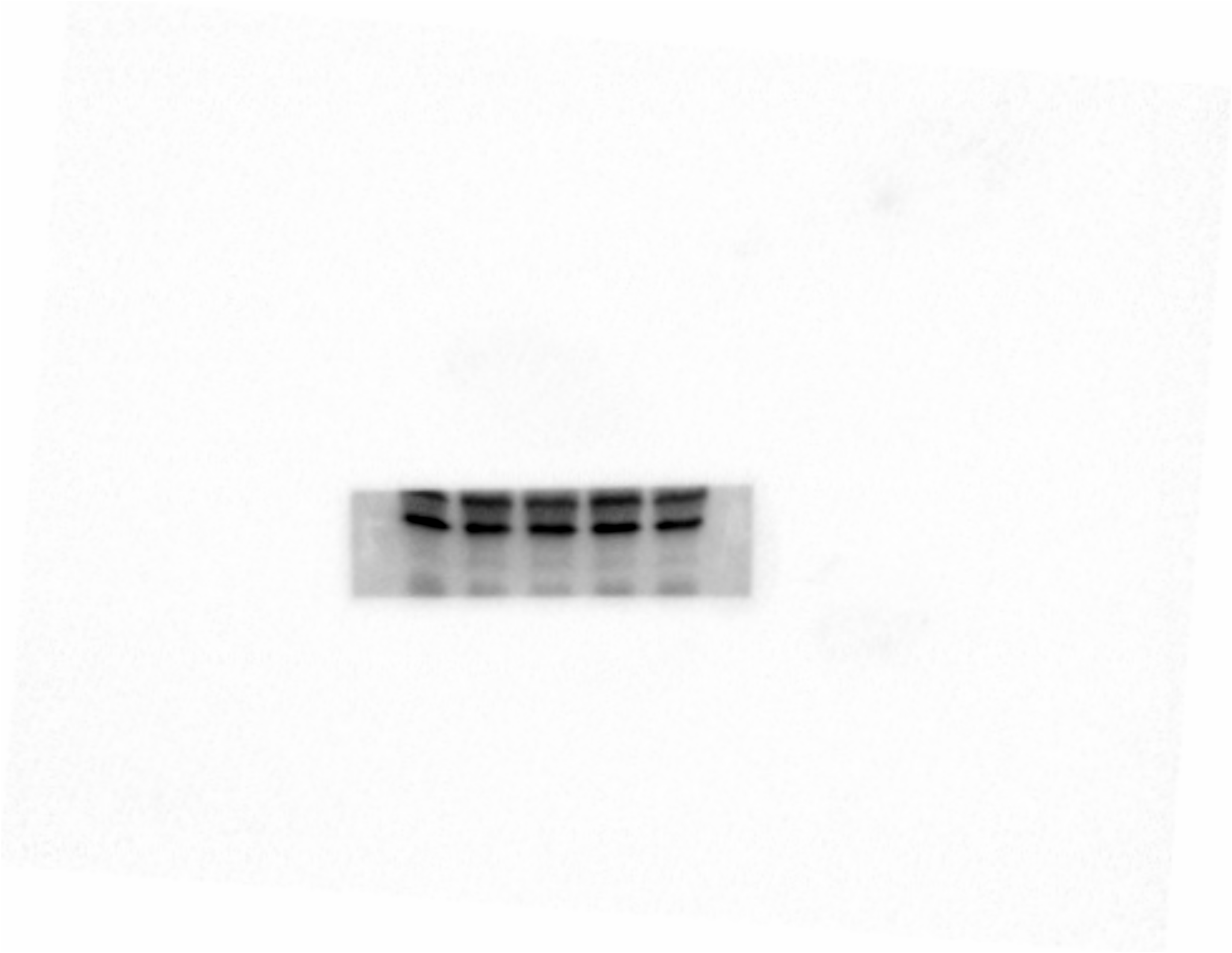

Supplement: Figure 5—source data 2. [file elife-96600-fig5-data2.zip › Raw unedited gels for Figure 5A/β-Actin-Cleaved Casepase 3.tif]

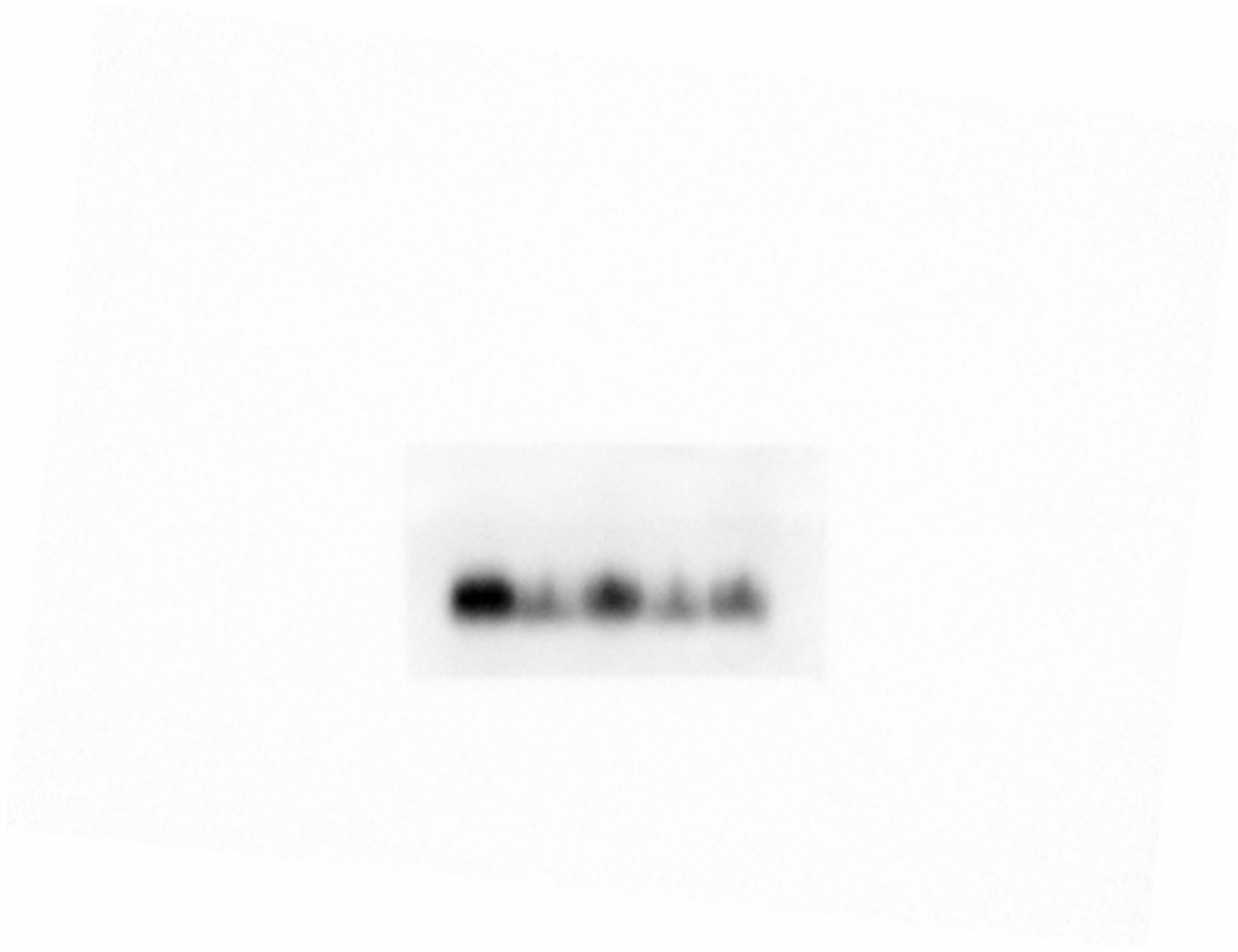

Supplement: Figure 6—source data 2. [file elife-96600-fig6-data2.zip › Raw unedited gels for Figure 6B and 6F/INSULIN.tif]

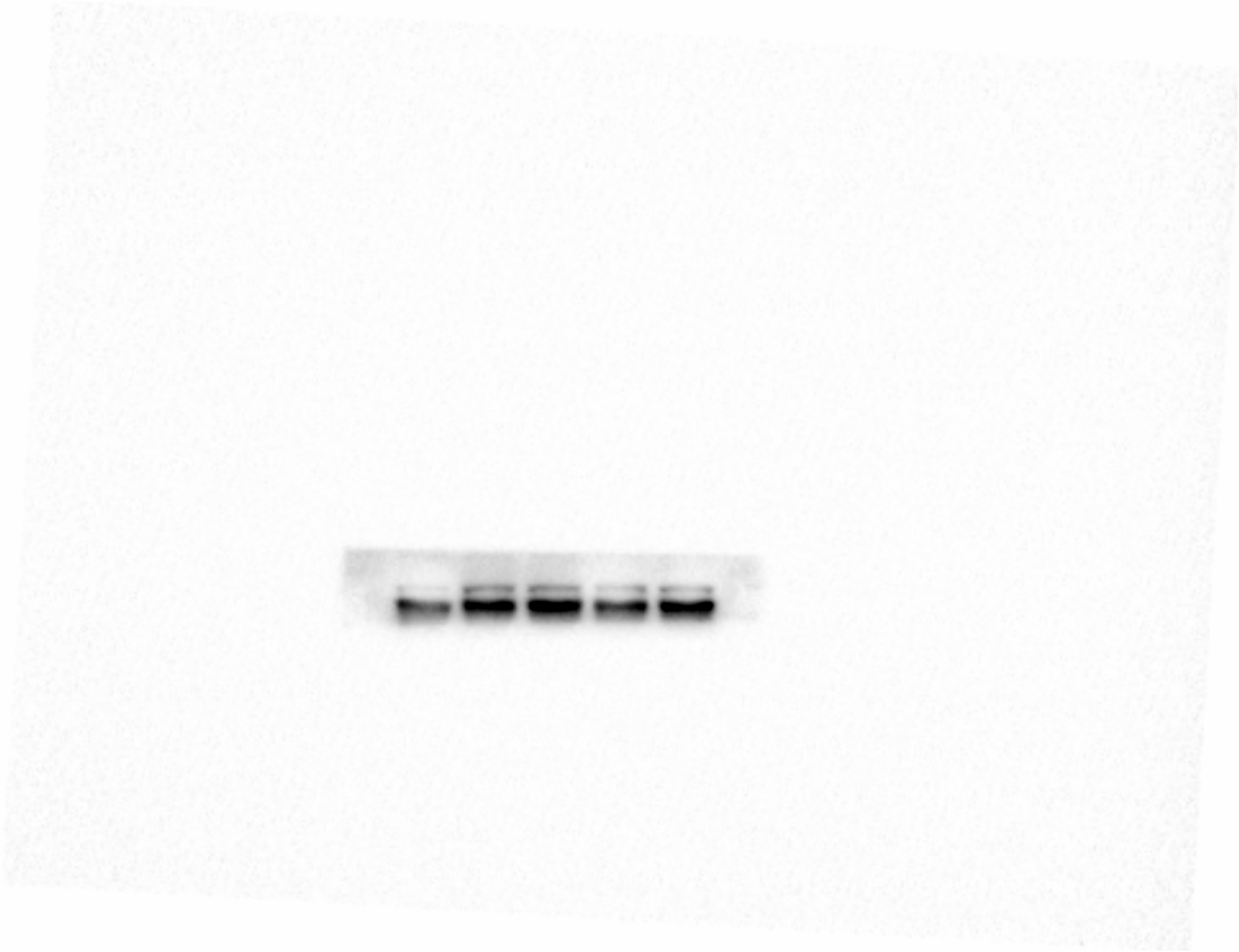

Supplement: Figure 6—source data 2. [file elife-96600-fig6-data2.zip › Raw unedited gels for Figure 6B and 6F/N-NRF2.tif]

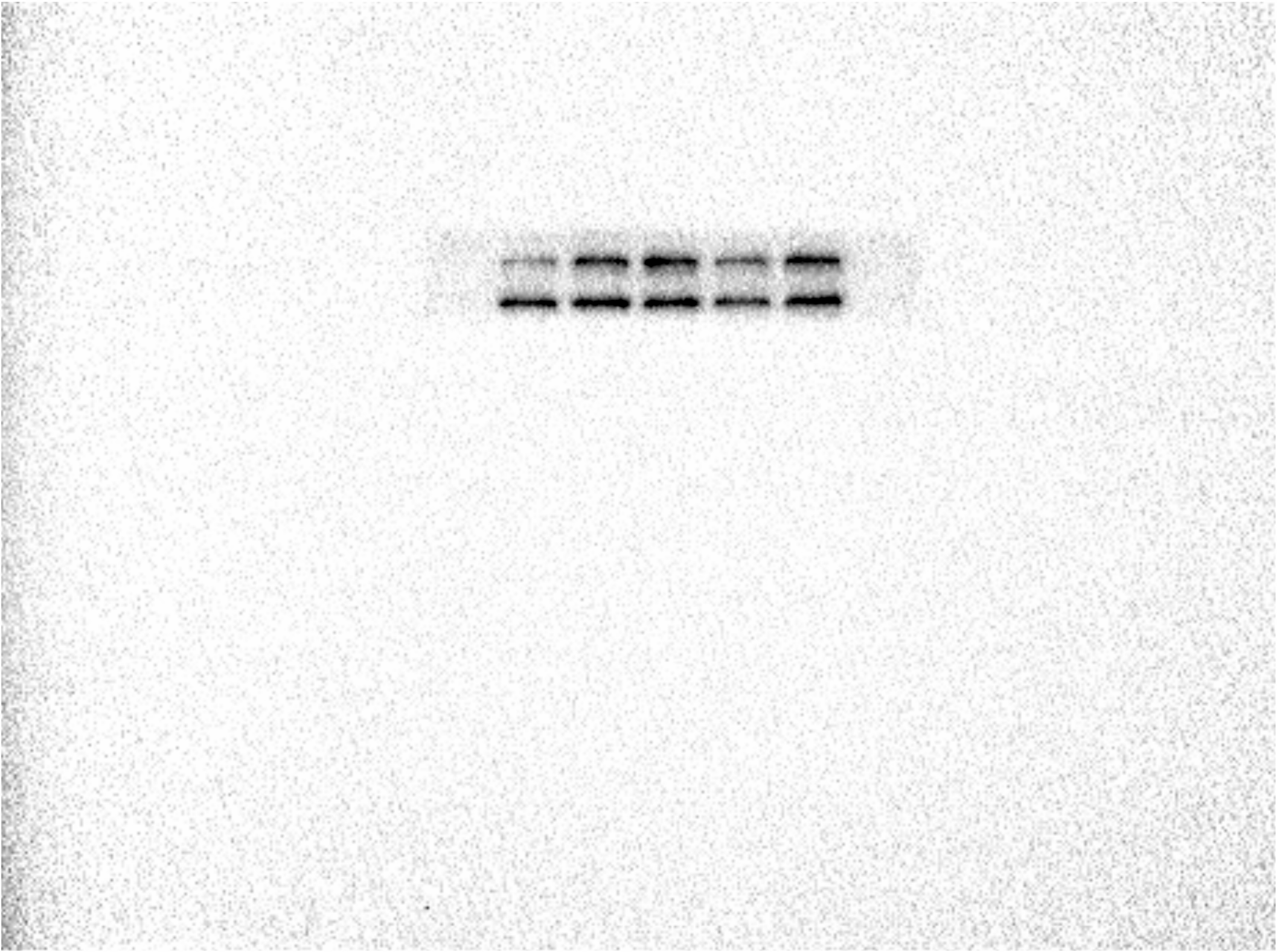

Supplement: Figure 6—source data 2. [file elife-96600-fig6-data2.zip › Raw unedited gels for Figure 6B and 6F/T-NRF2.tif]

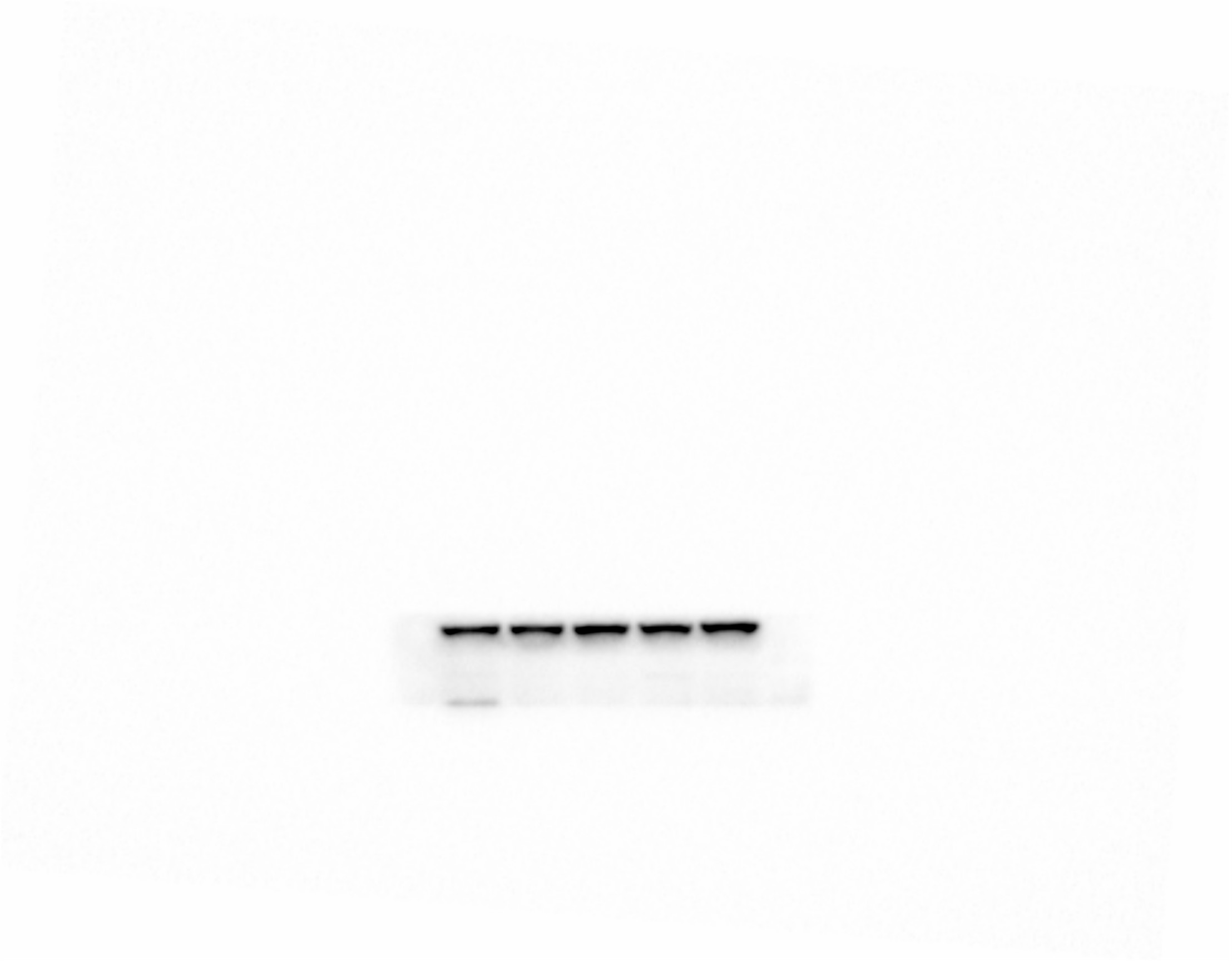

Supplement: Figure 6—source data 2. [file elife-96600-fig6-data2.zip › Raw unedited gels for Figure 6B and 6F/β-Actin-N-NRF2.tif]

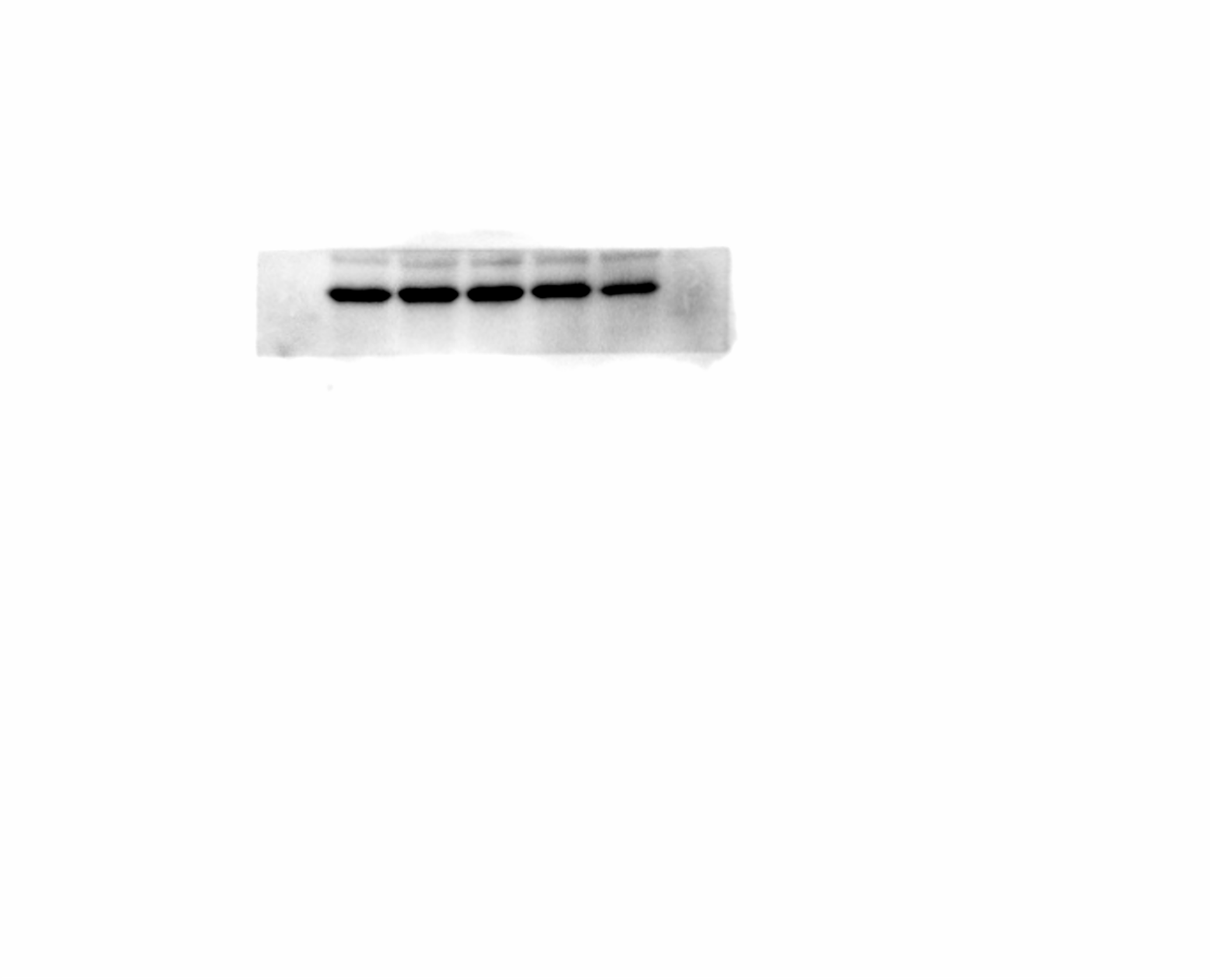

Supplement: Figure 6—source data 2. [file elife-96600-fig6-data2.zip › Raw unedited gels for Figure 6B and 6F/β-Actin-T-NRF2.tif]

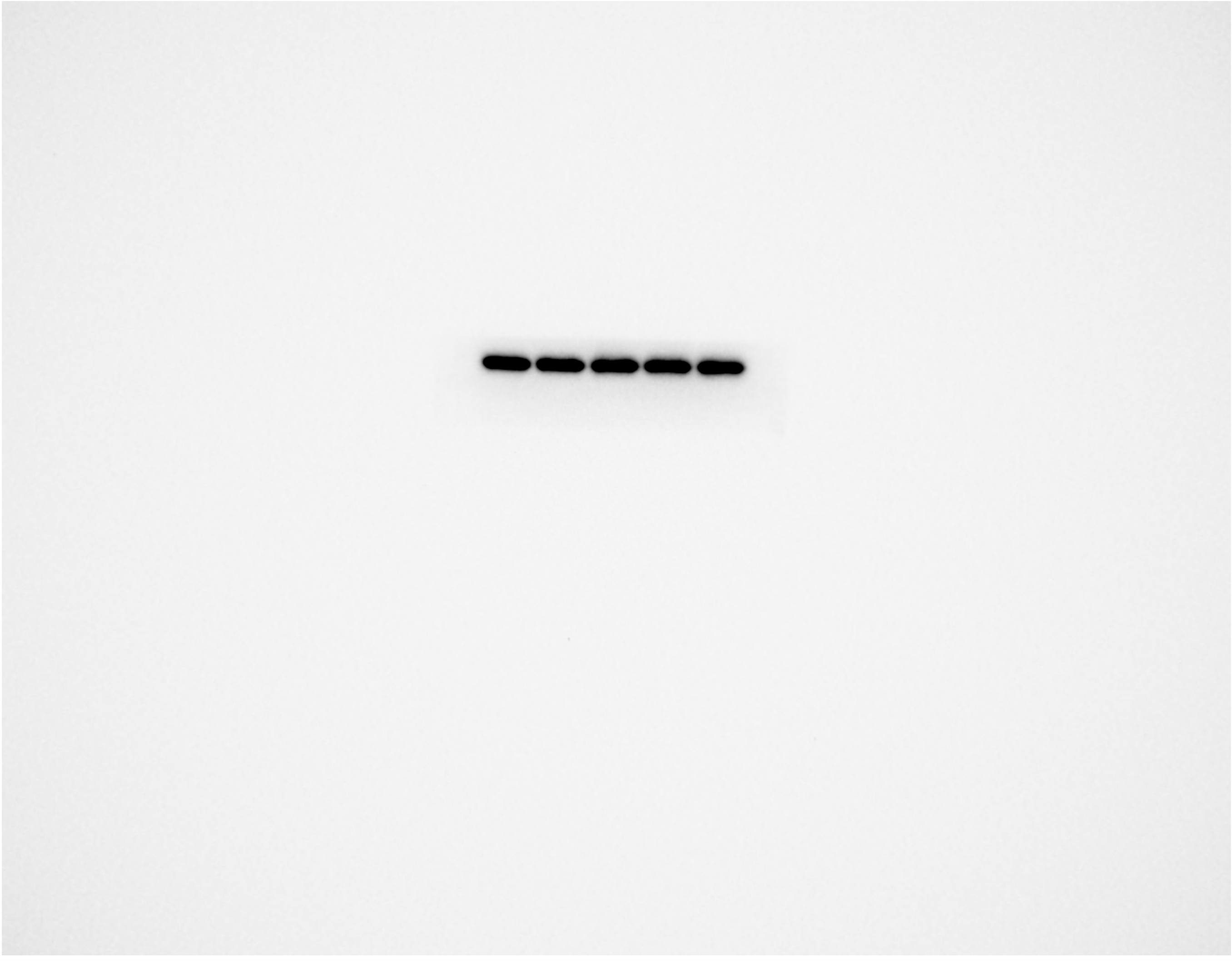

Supplement: Figure 6—source data 2. [file elife-96600-fig6-data2.zip › Raw unedited gels for Figure 6B and 6F/β-Actin.tif]

Full unedited gel for Figure 7A. The red box shows the image used in the manuscript.

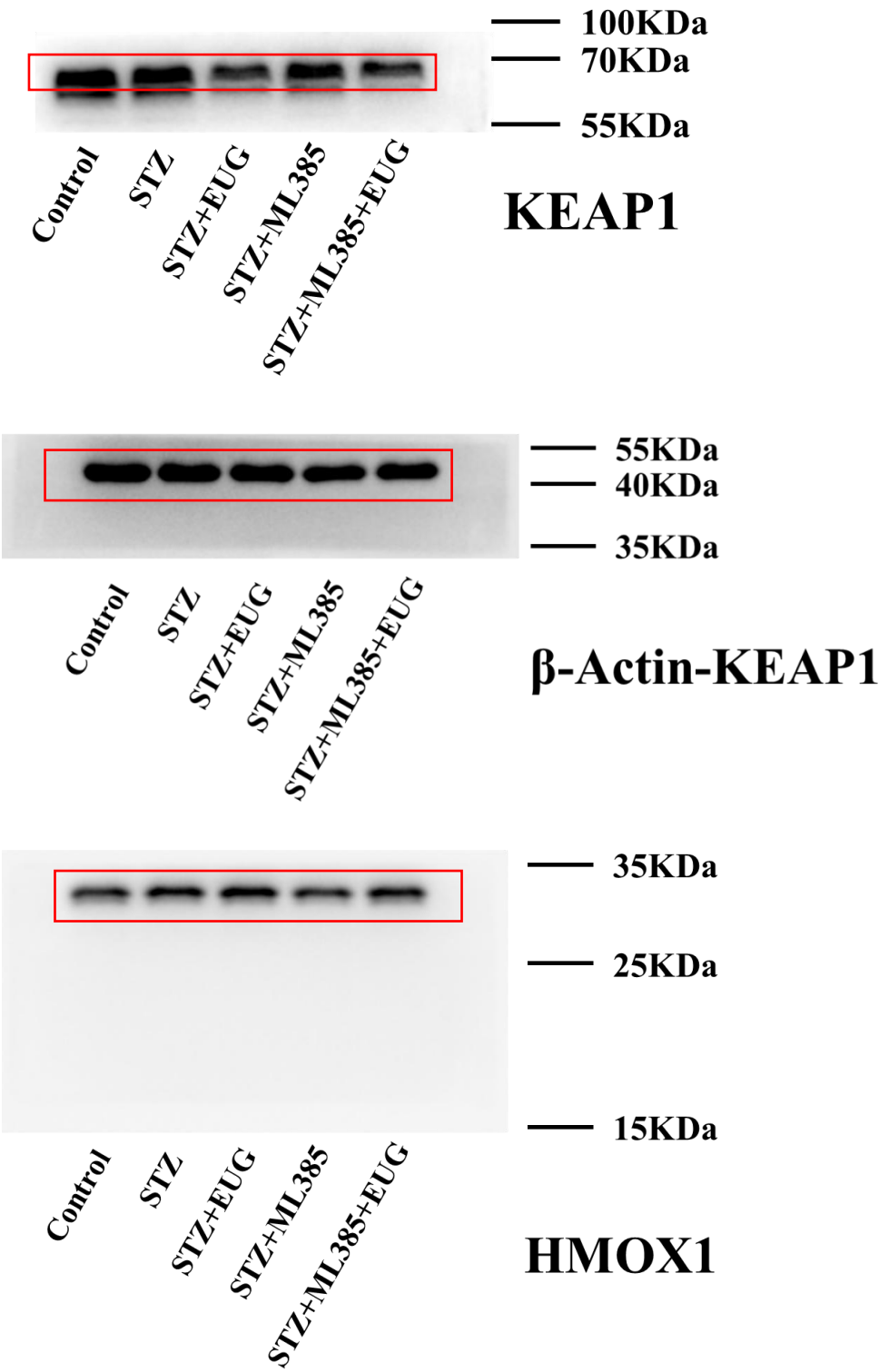

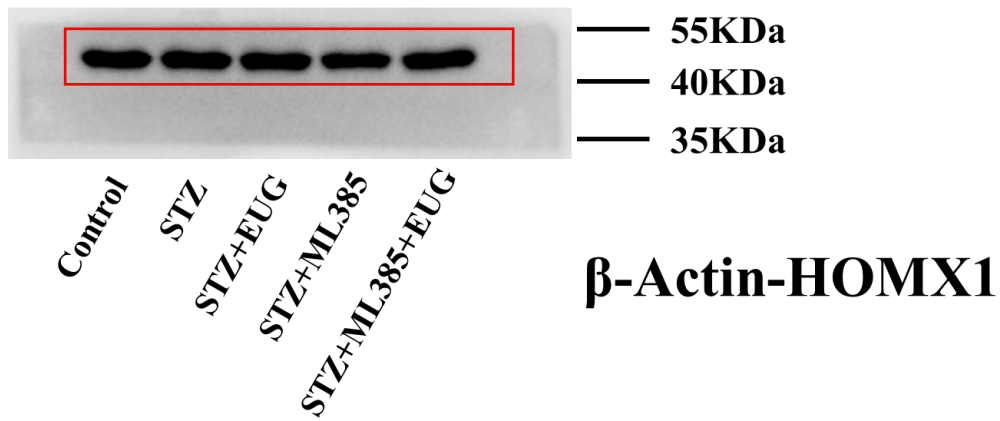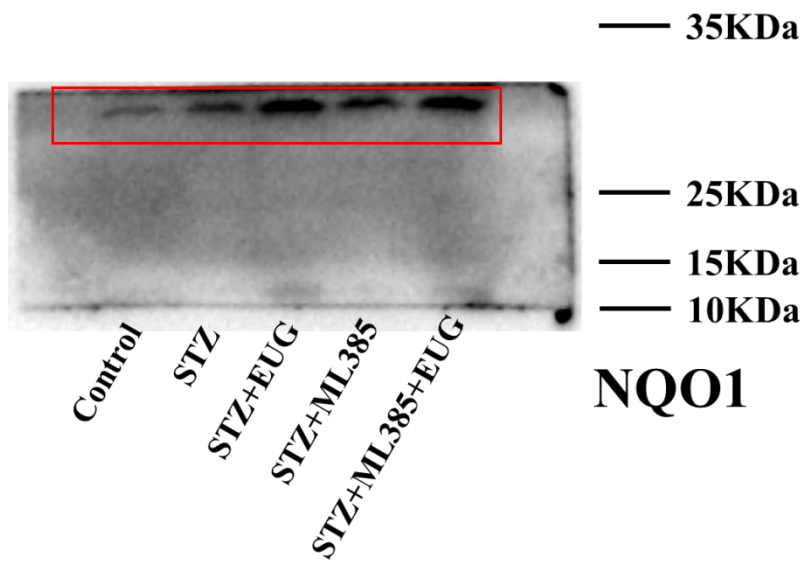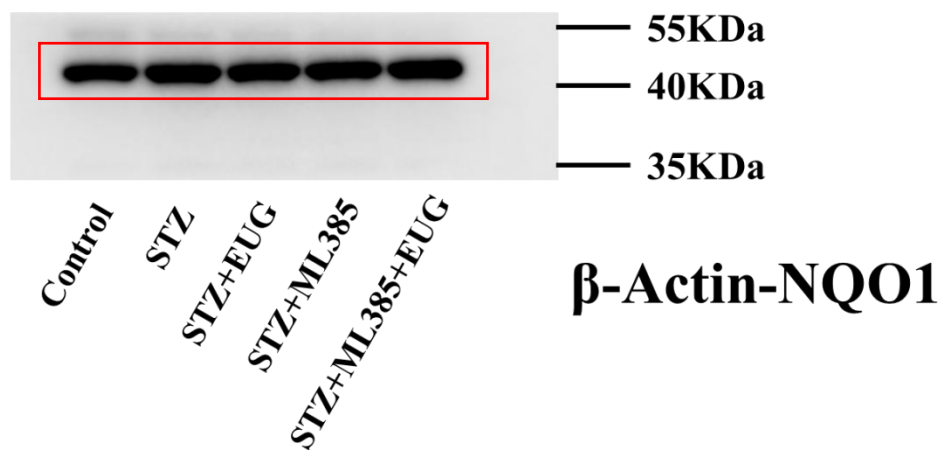

Supplement: Figure 7—source data 1. [file elife-96600-fig7-data1.pdf]

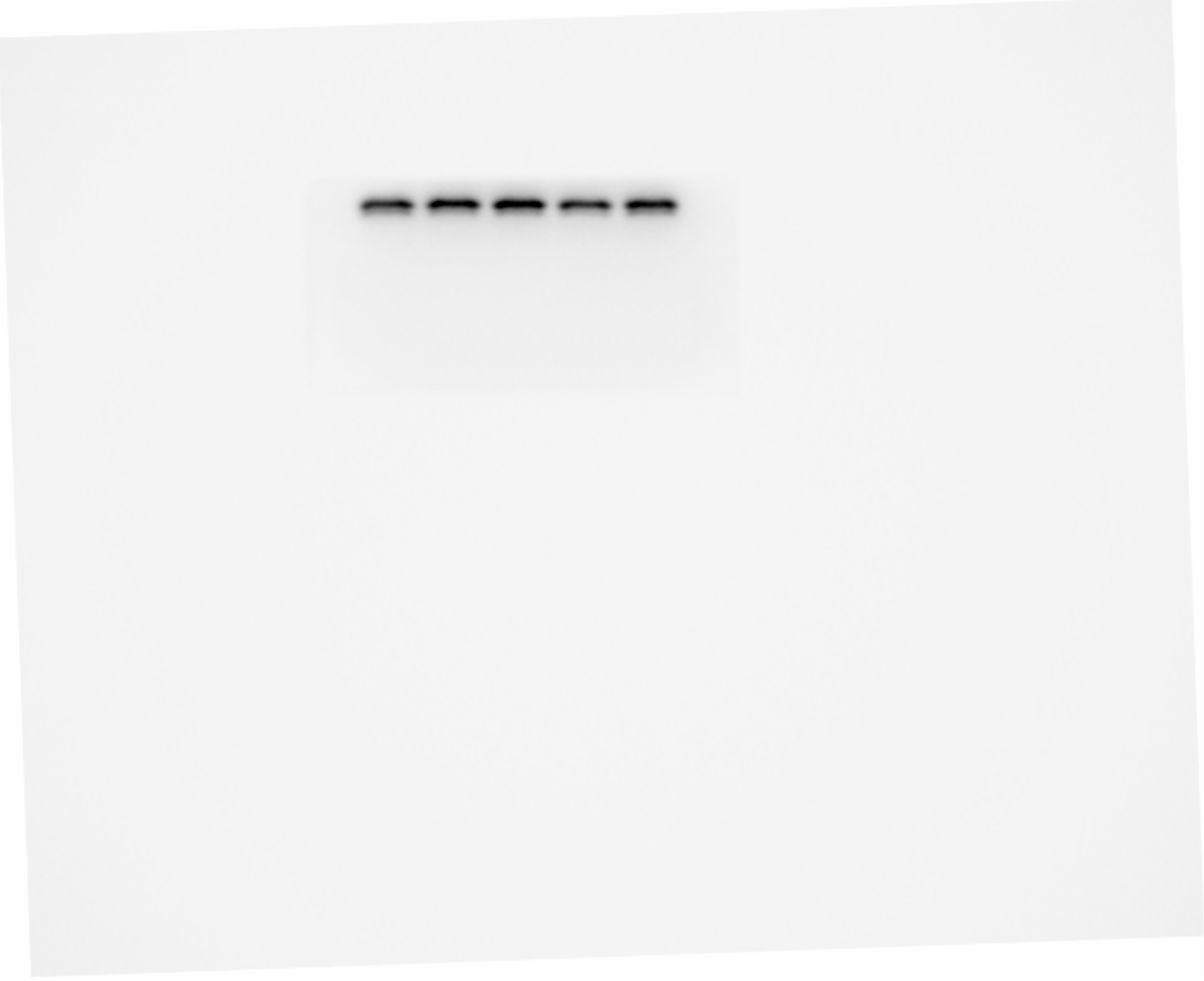

Supplement: Figure 7—source data 2. [file elife-96600-fig7-data2.zip › Raw unedited gels for Figure 7A/HMOX1.tif]

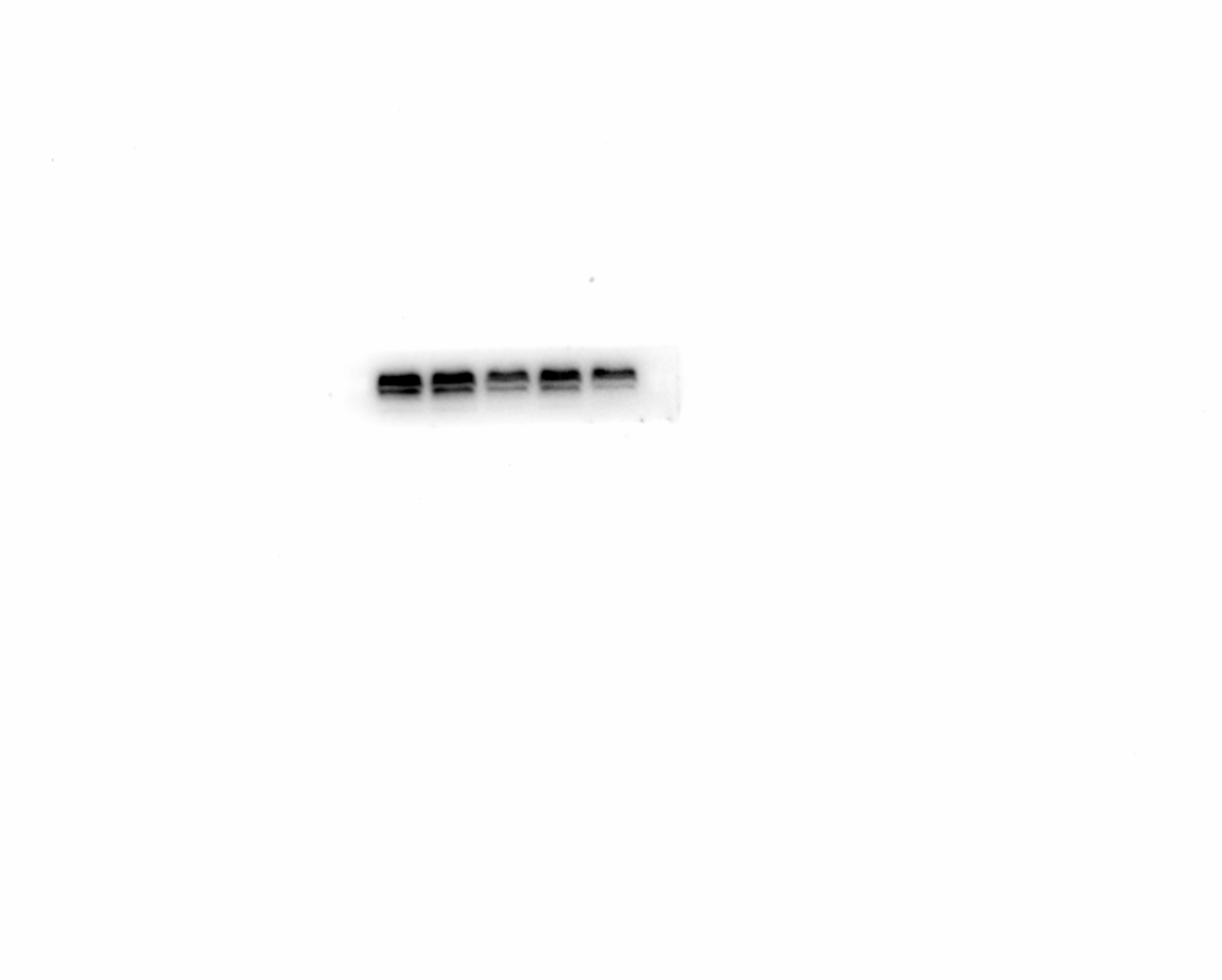

Supplement: Figure 7—source data 2. [file elife-96600-fig7-data2.zip › Raw unedited gels for Figure 7A/KEAP1.tif]

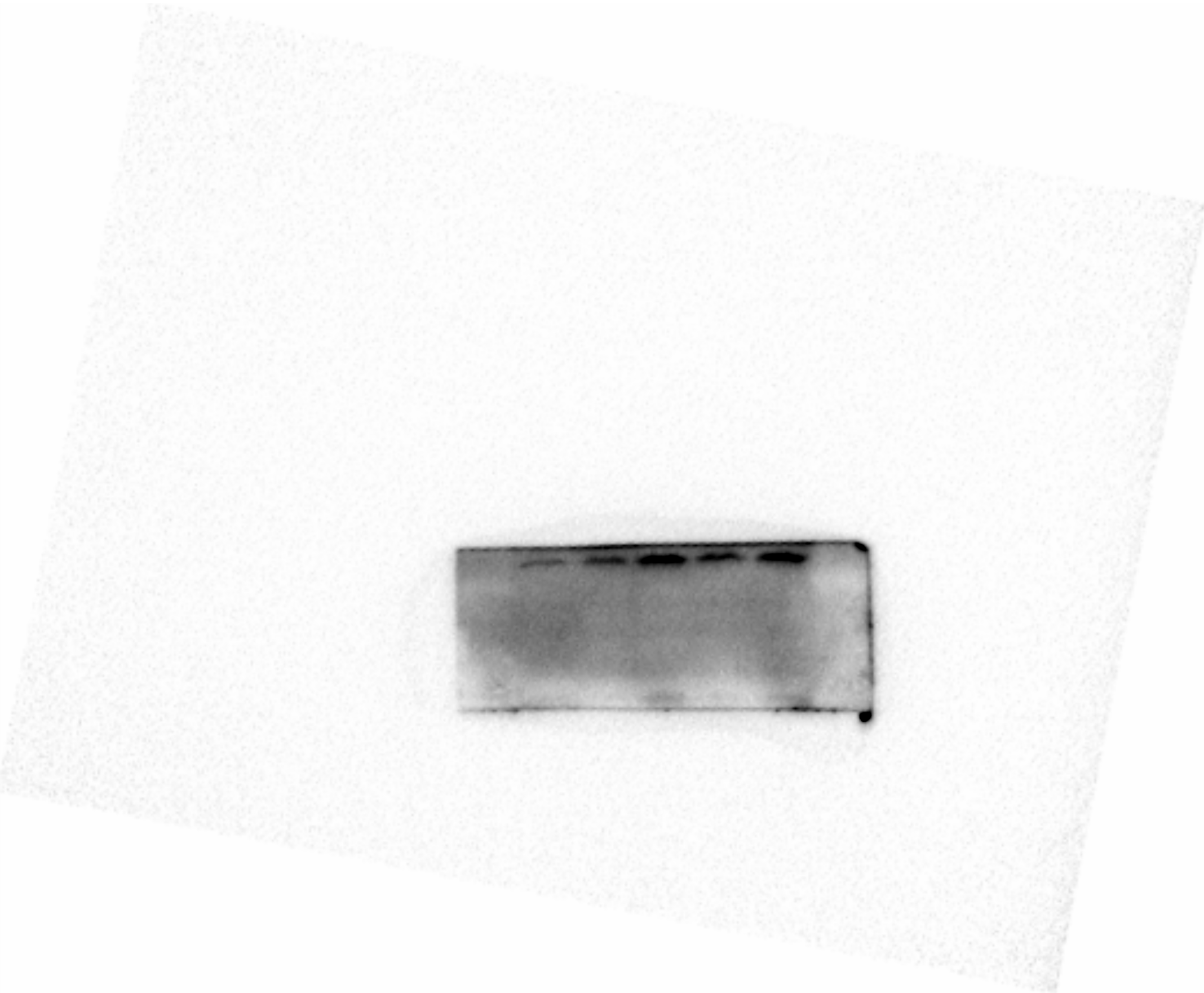

Supplement: Figure 7—source data 2. [file elife-96600-fig7-data2.zip › Raw unedited gels for Figure 7A/NQO1.tif]

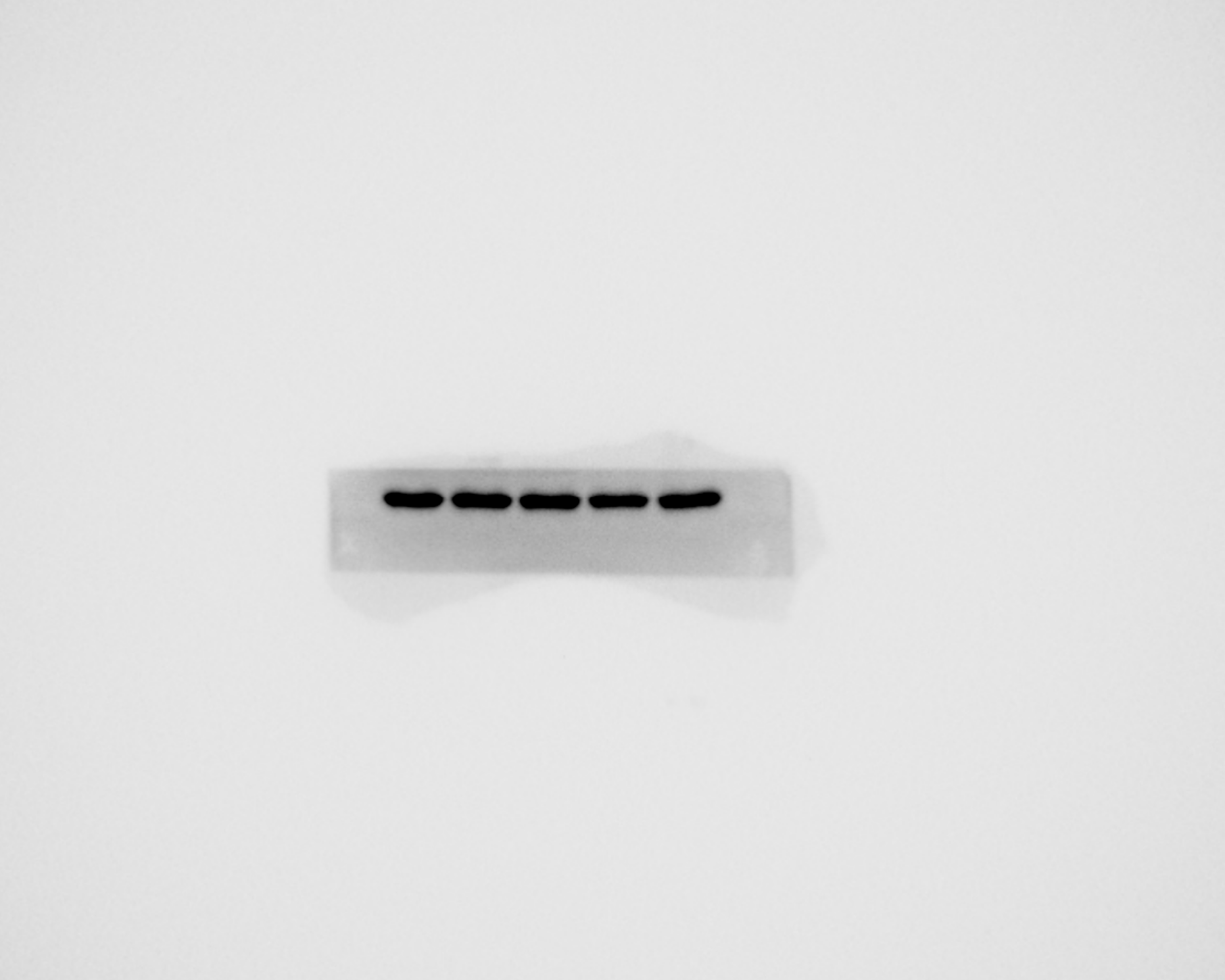

Supplement: Figure 7—source data 2. [file elife-96600-fig7-data2.zip › Raw unedited gels for Figure 7A/β-Actin-HMOX1.tif]

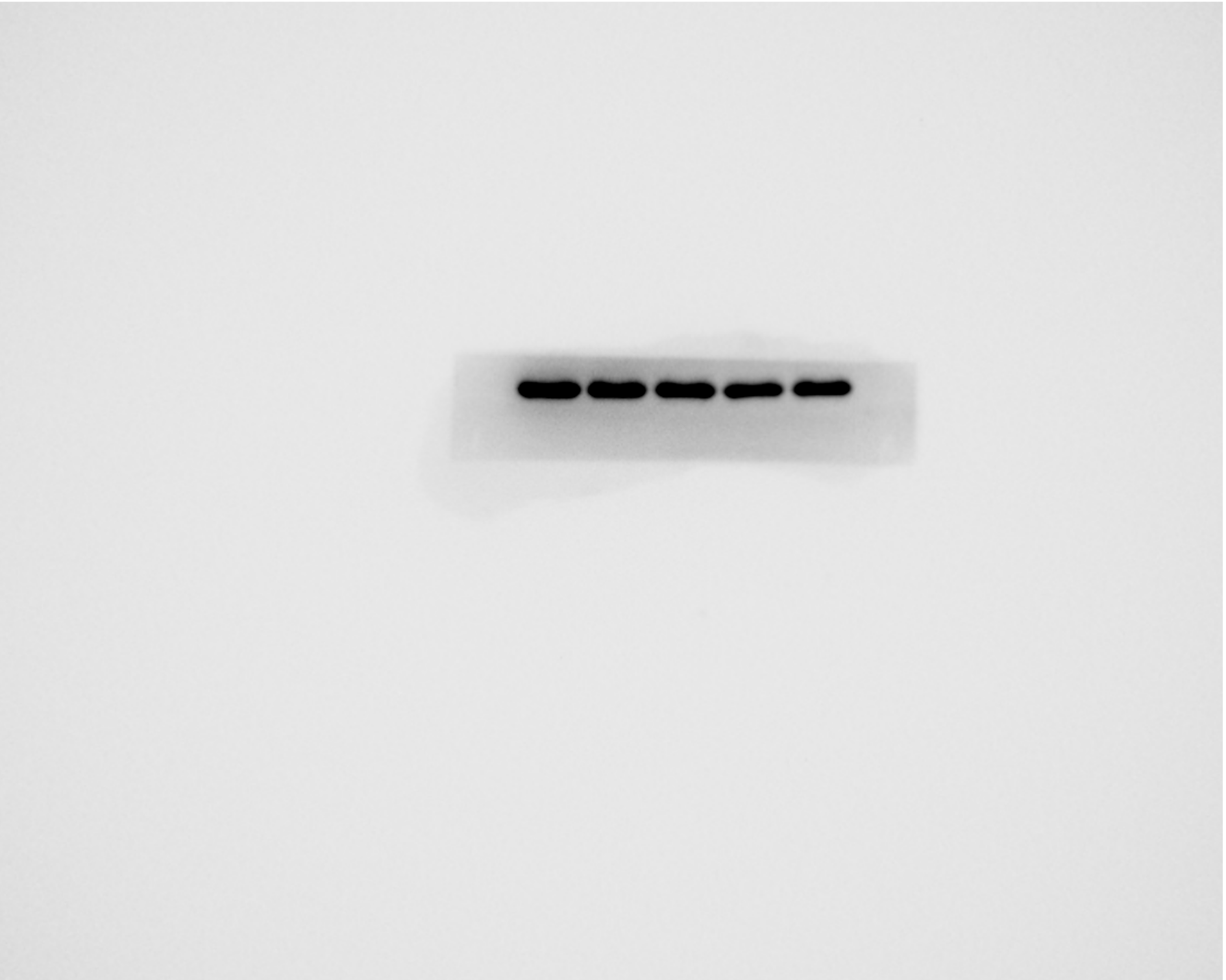

Supplement: Figure 7—source data 2. [file elife-96600-fig7-data2.zip › Raw unedited gels for Figure 7A/β-Actin-KEAP1.tif]

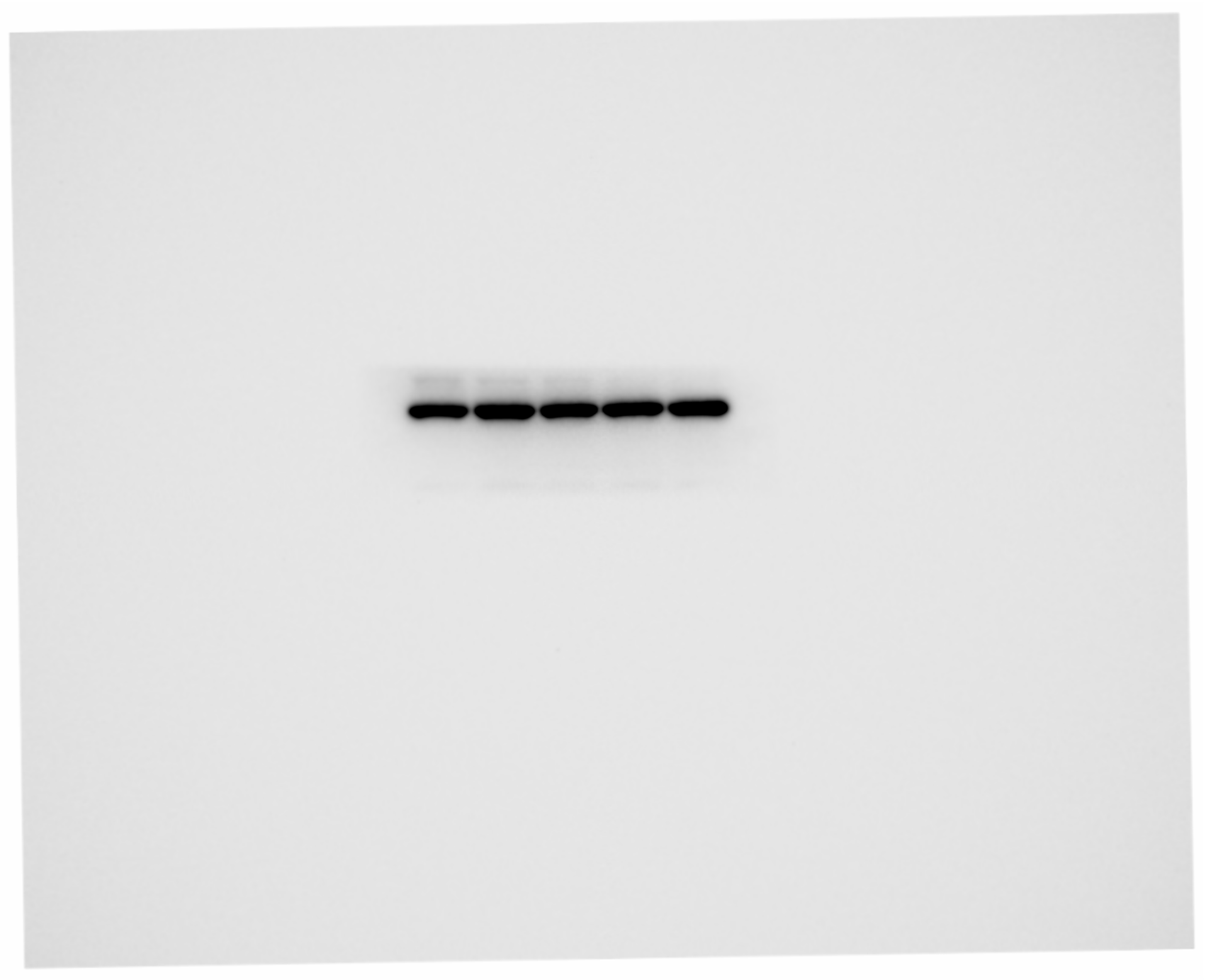

Supplement: Figure 7—source data 2. [file elife-96600-fig7-data2.zip › Raw unedited gels for Figure 7A/β-Actin-NQO1.tif]

Full unedited gel for Figure 8A. The red box shows the image used in the manuscript.

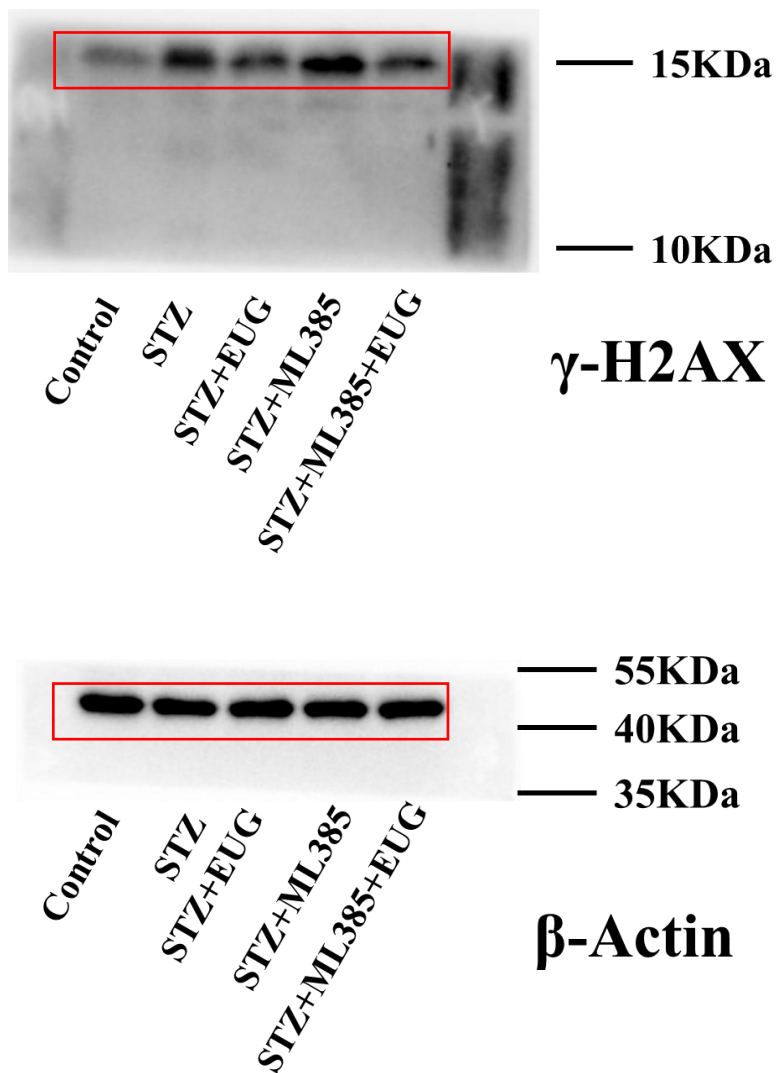

Supplement: Figure 8—source data 1. [file elife-96600-fig8-data1.pdf]

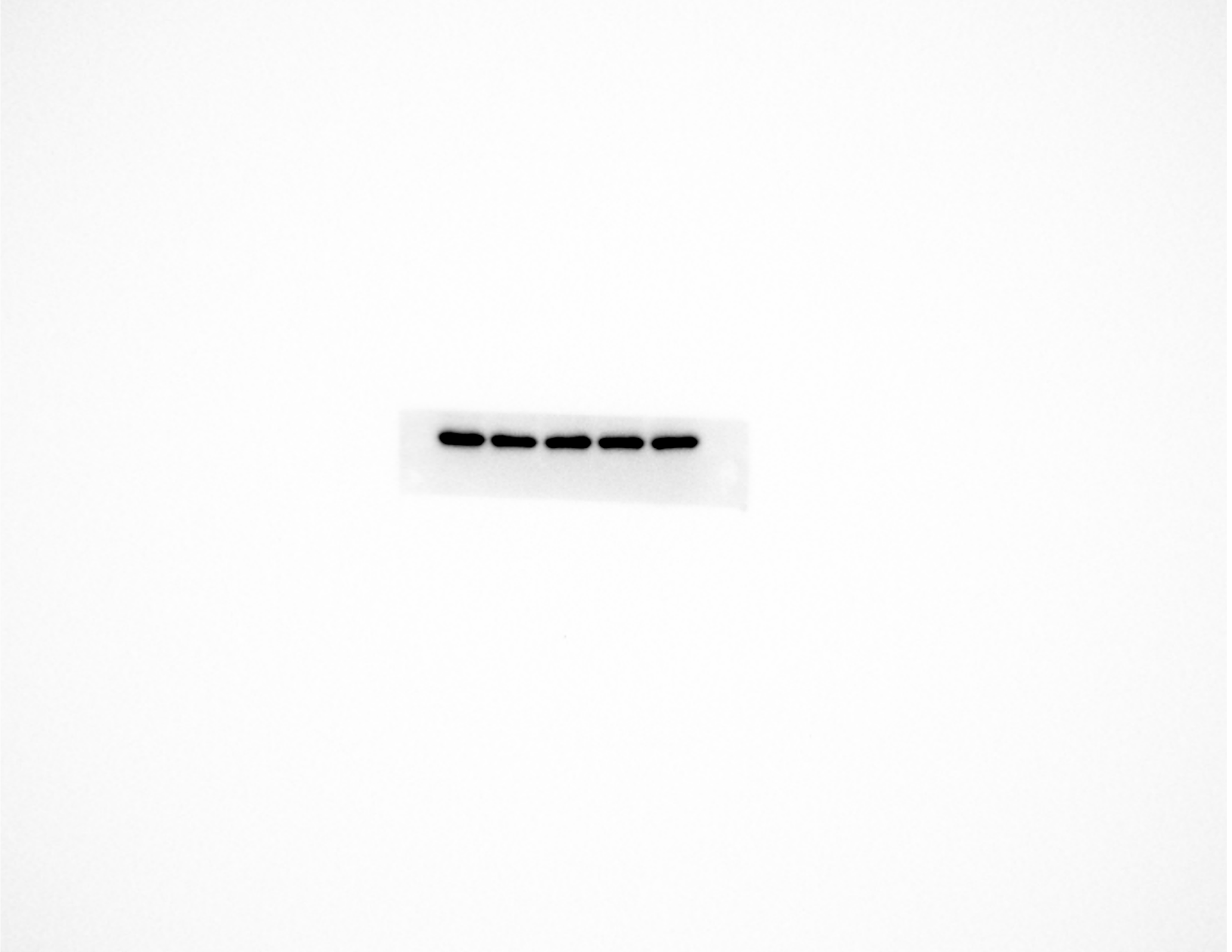

Supplement: Figure 8—source data 2. [file elife-96600-fig8-data2.zip › Raw unedited gels for Figure 8A/β-Actin.tif]

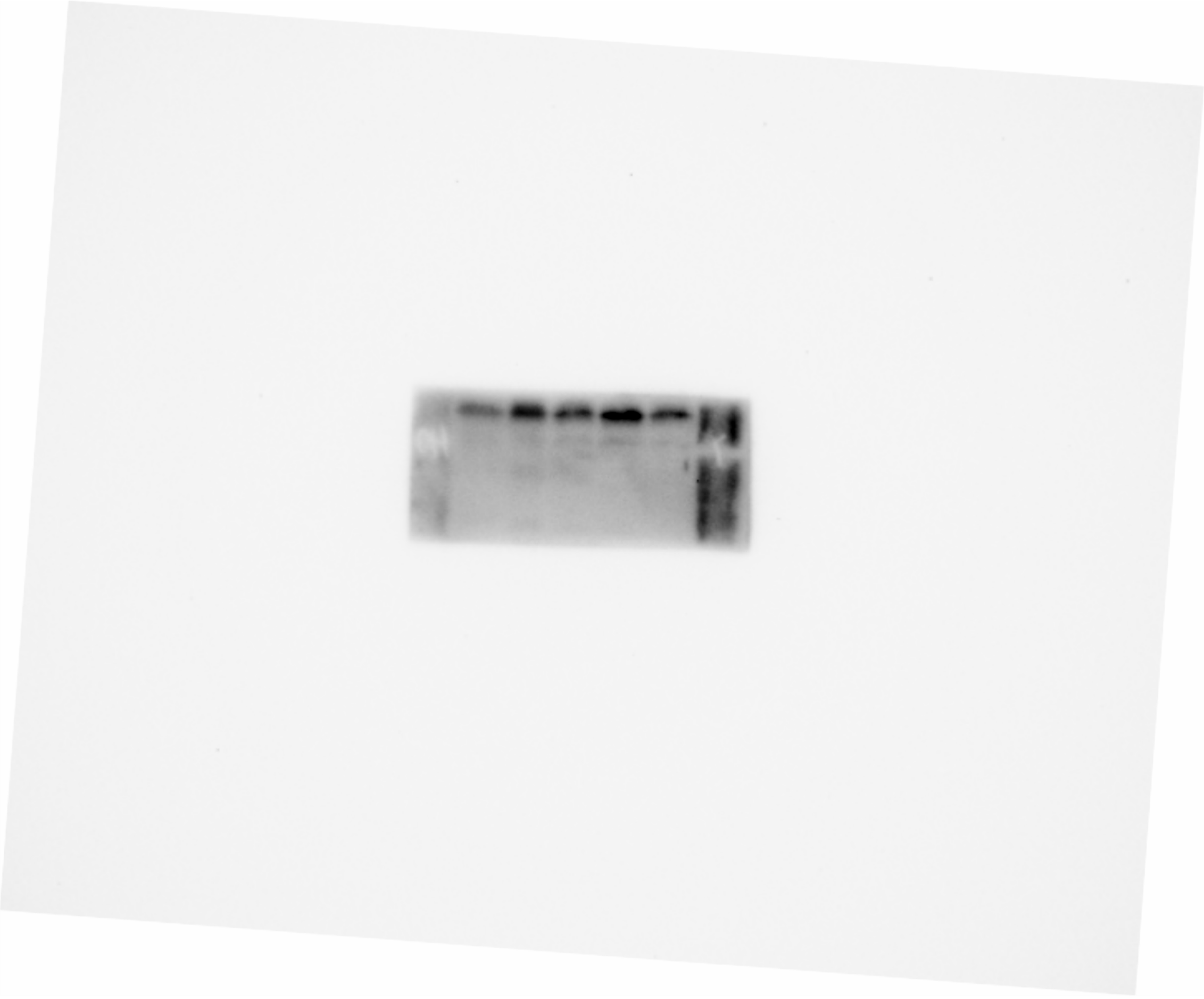

Supplement: Figure 8—source data 2. [file elife-96600-fig8-data2.zip › Raw unedited gels for Figure 8A/γH2AX.tif]

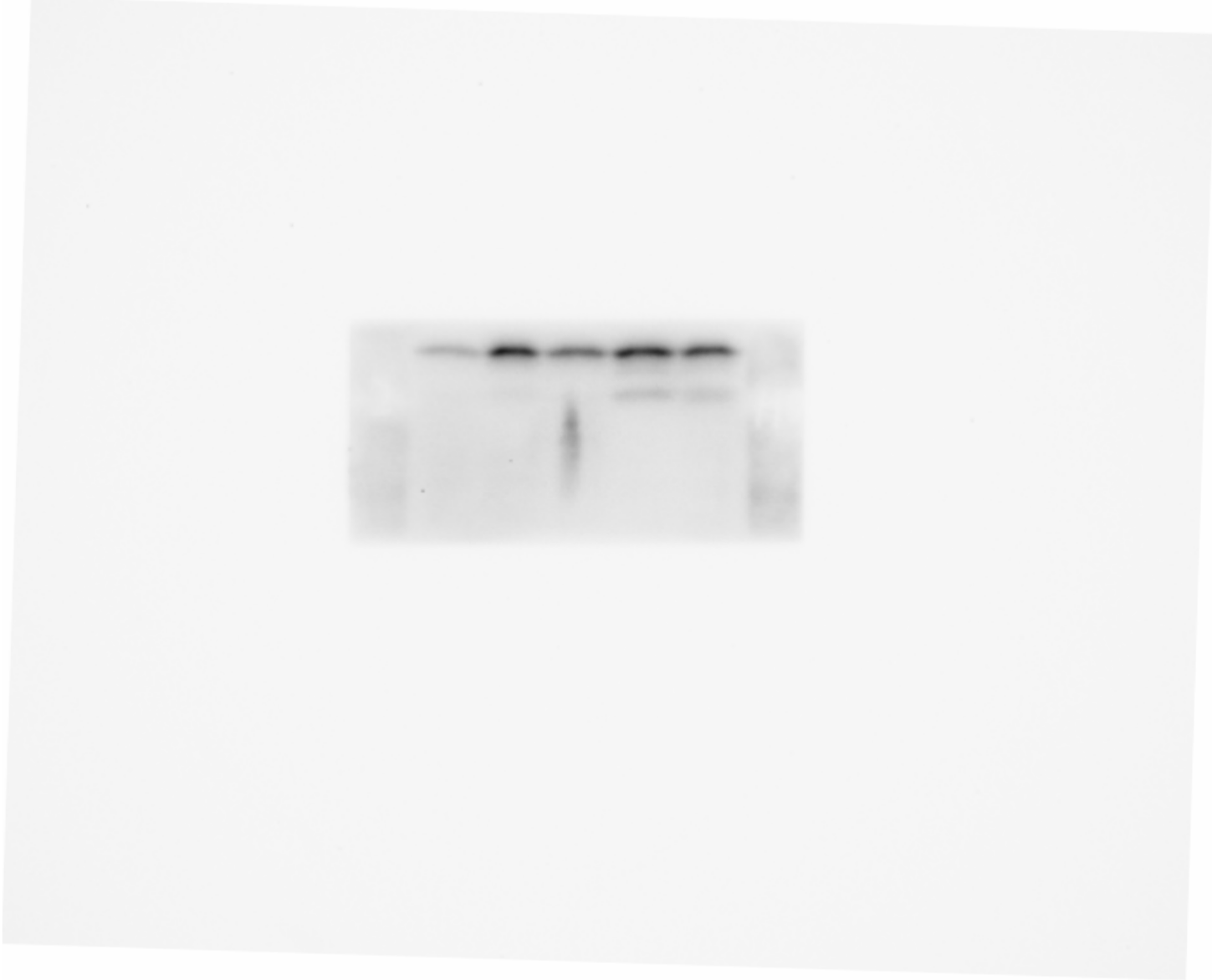

Supplement: Figure 9—source data 2. [file elife-96600-fig9-data2.zip › Raw unedited gels for Figure 9A/BAX.tif]

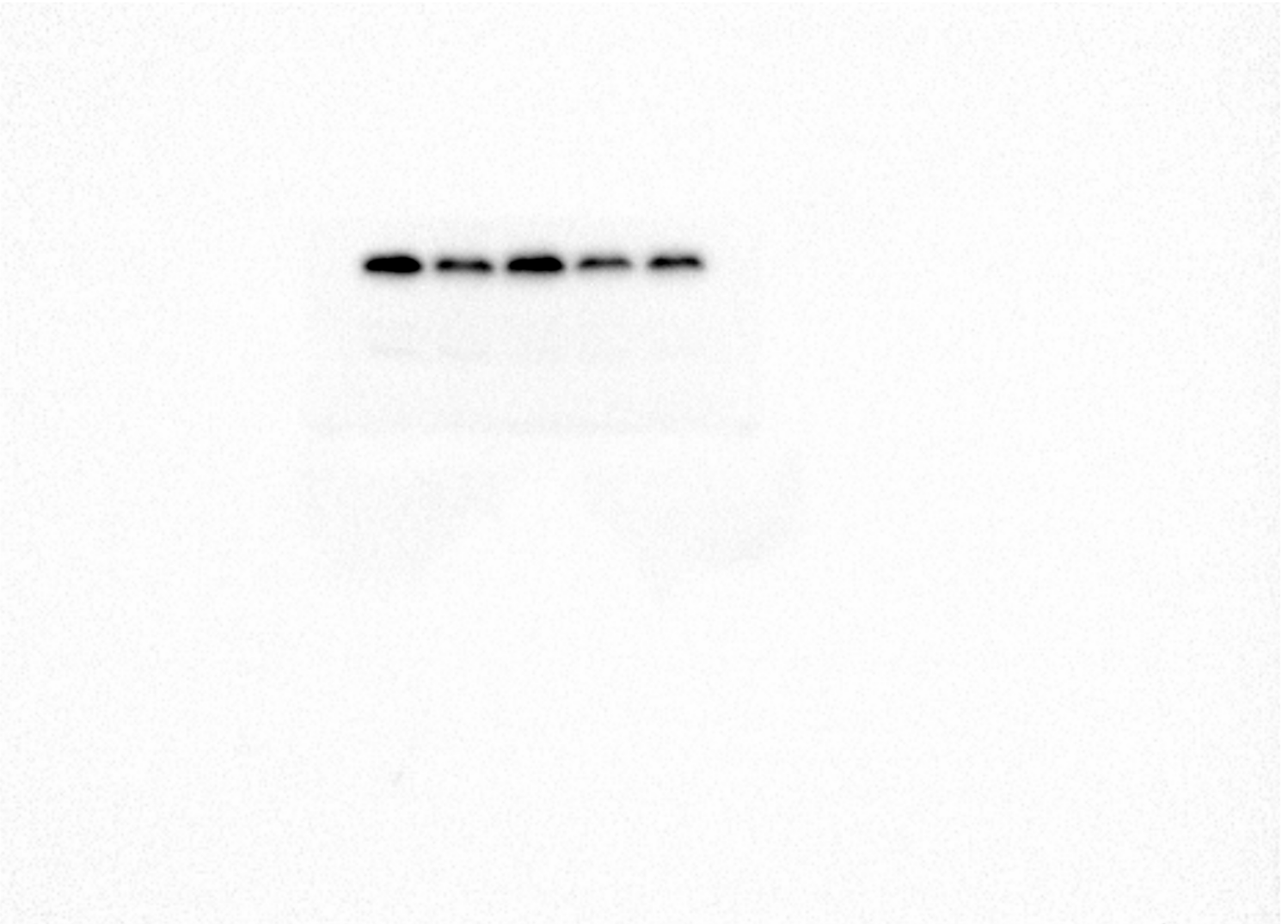

Supplement: Figure 9—source data 2. [file elife-96600-fig9-data2.zip › Raw unedited gels for Figure 9A/BCL2.tif]

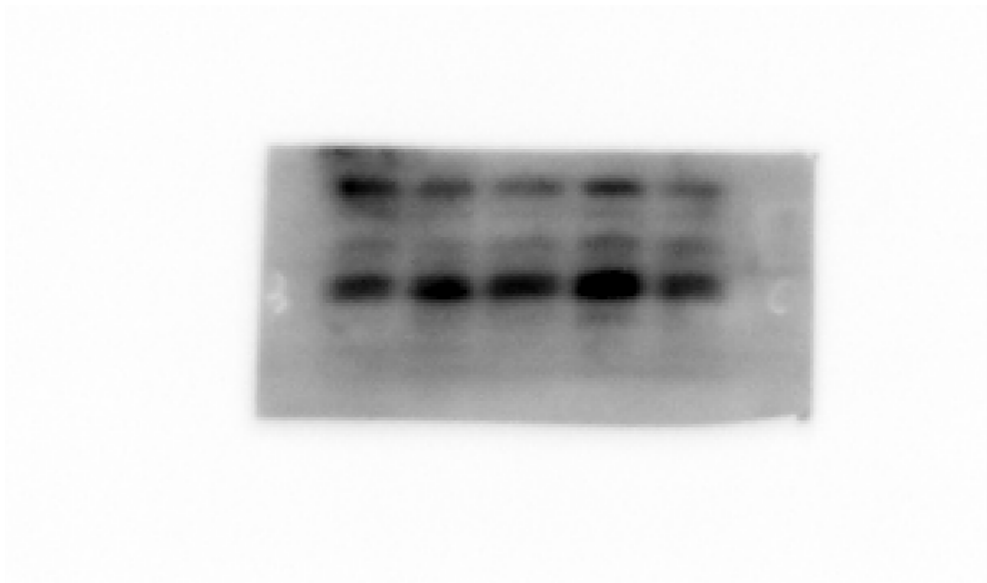

Supplement: Figure 9—source data 2. [file elife-96600-fig9-data2.zip › Raw unedited gels for Figure 9A/Cleaved Casepase3.tif]

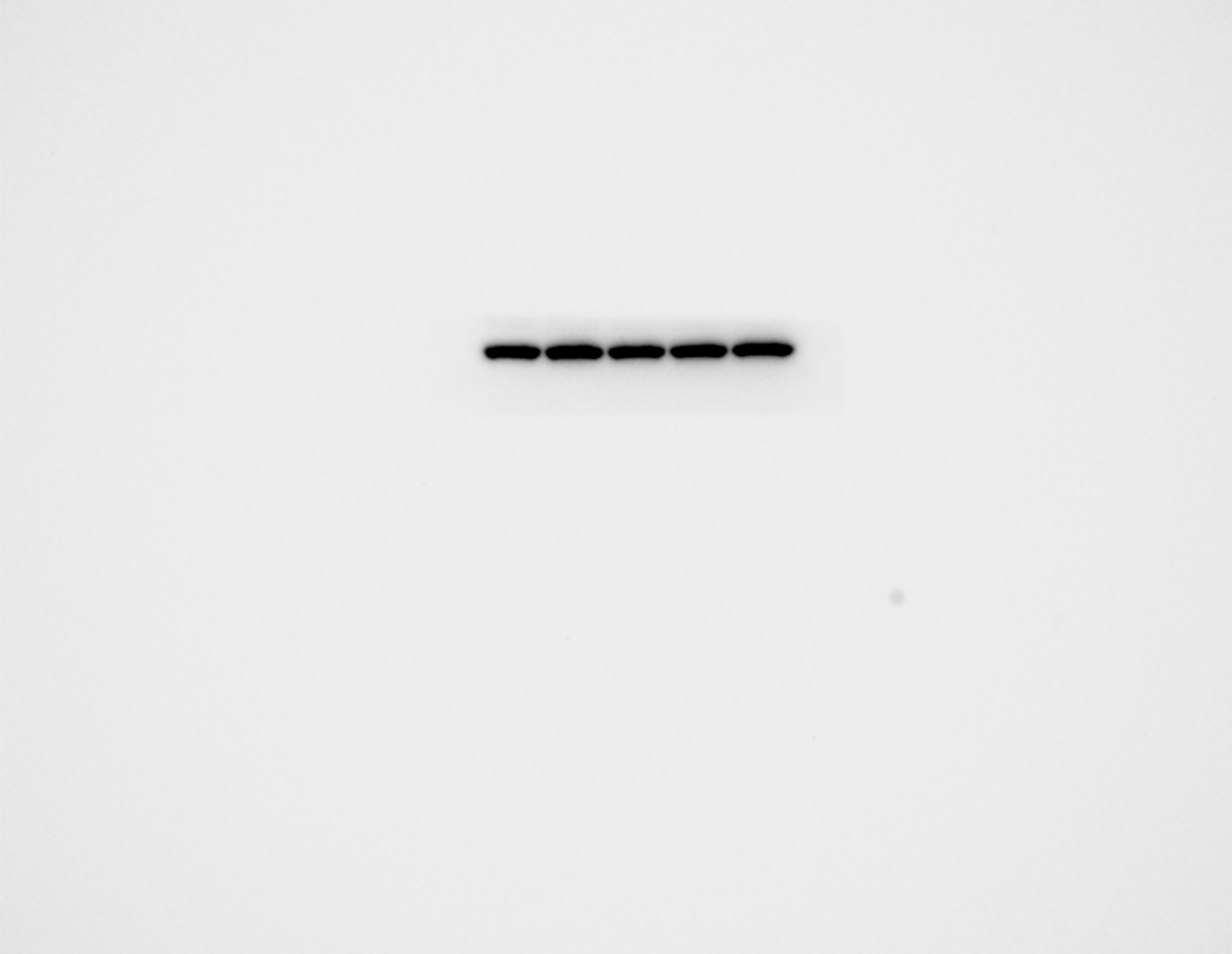

Supplement: Figure 9—source data 2. [file elife-96600-fig9-data2.zip › Raw unedited gels for Figure 9A/β-Actin-BAX.tif]

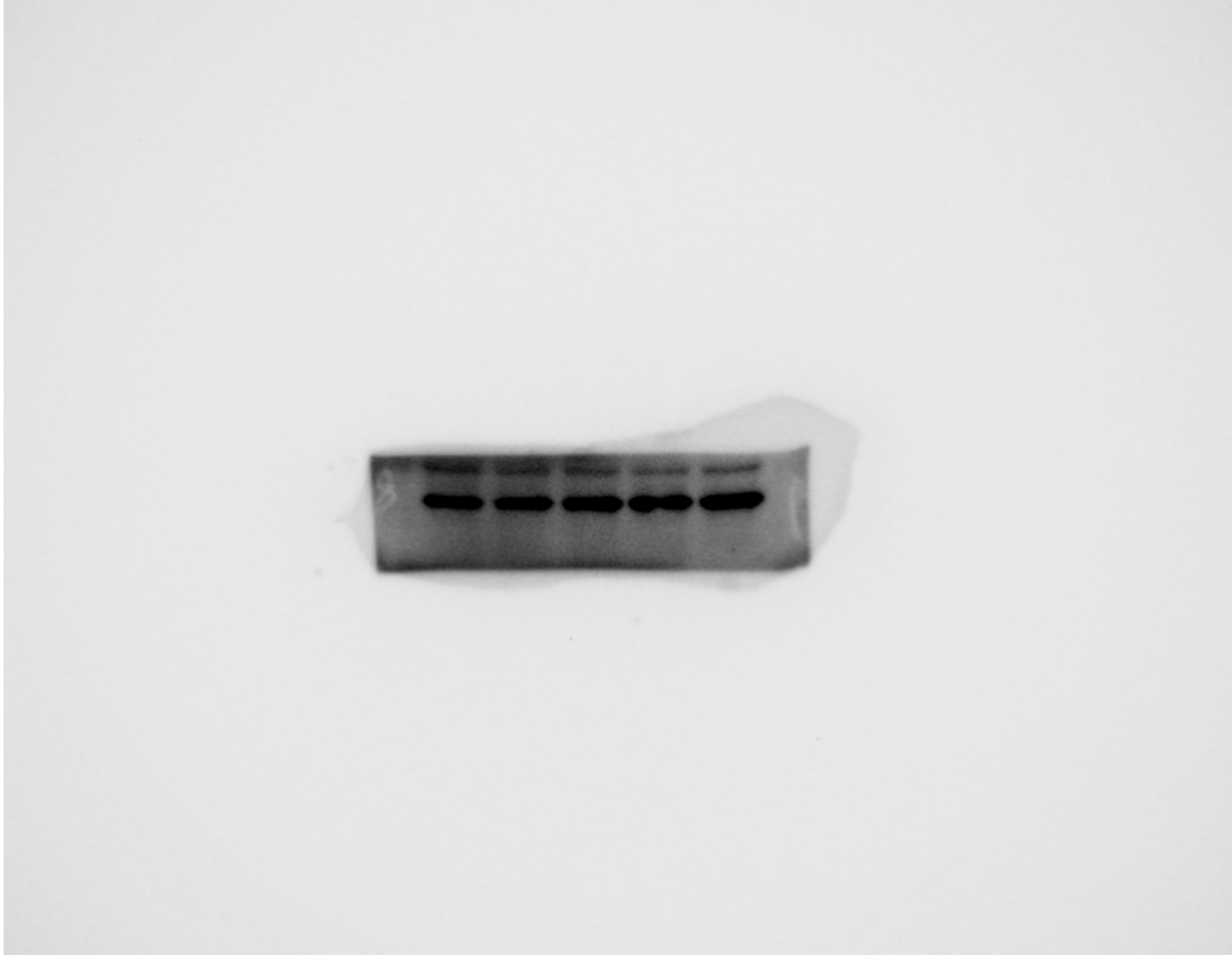

Supplement: Figure 9—source data 2. [file elife-96600-fig9-data2.zip › Raw unedited gels for Figure 9A/β-Actin-BCL2.tif]

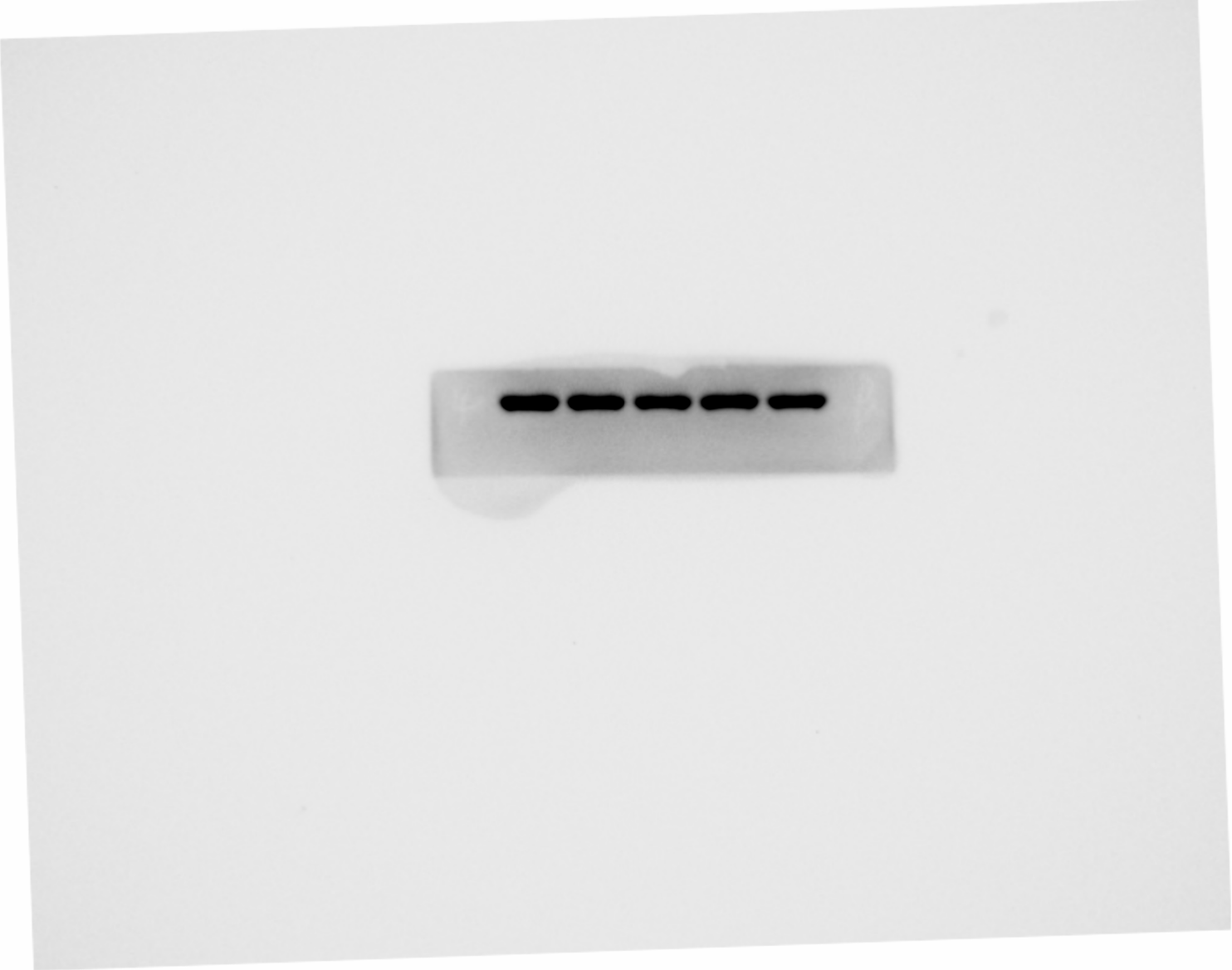

Supplement: Figure 9—source data 2. [file elife-96600-fig9-data2.zip › Raw unedited gels for Figure 9A/β-Actin-Cleaved Casepase 3.tif]
