# Supplementary material for: The protective roles of eugenol on type 1 diabetes mellitus through NRF2-mediated oxidative stress pathway
Source: eLife. 2025 Jan 10;13:RP96600. doi: 10.7554/eLife.96600 (PMC11723580; doi:10.7554/eLife.96600)
Supplement: Figure 3—source data 1. [file elife-96600-fig3-data1.pdf]

Full unedited gel for Figure 3A. The red box shows the image used in the manuscript.

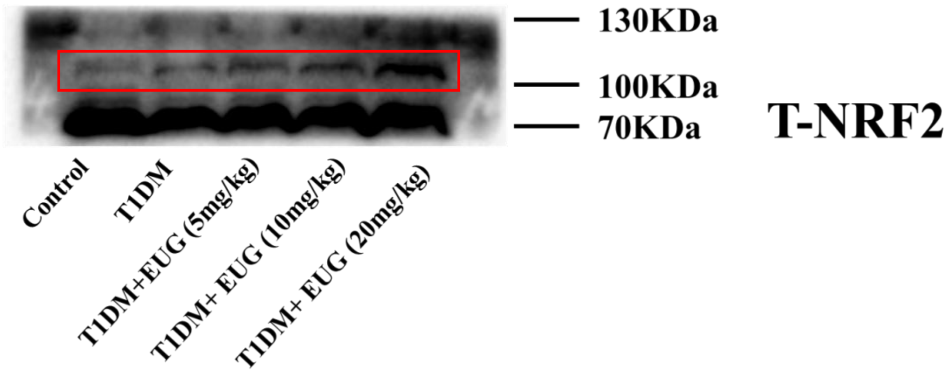

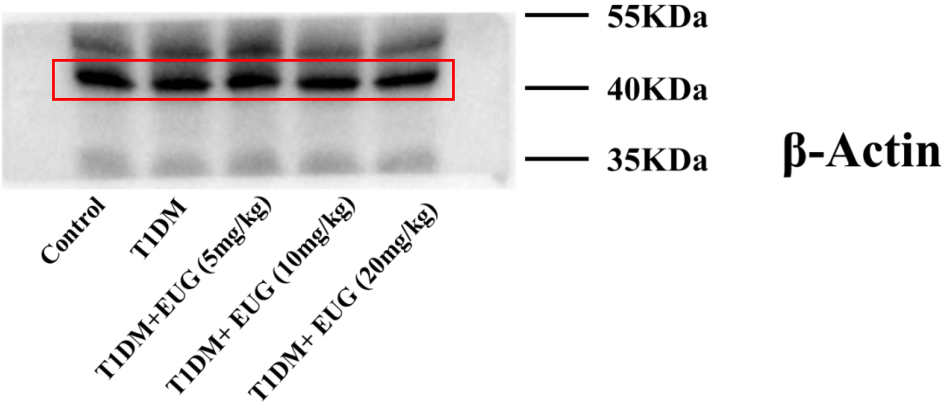

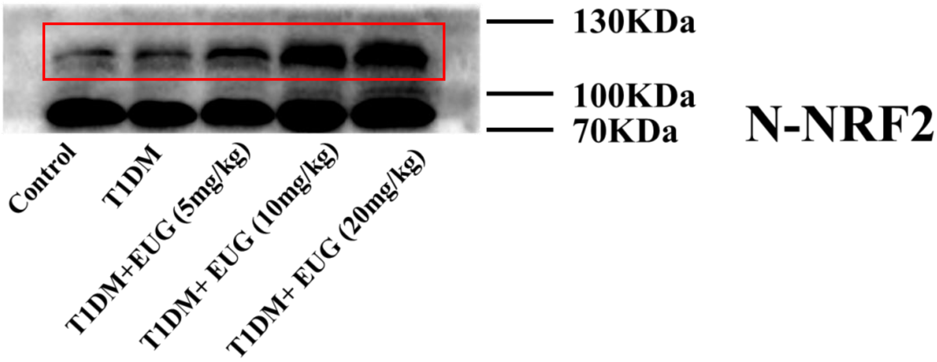

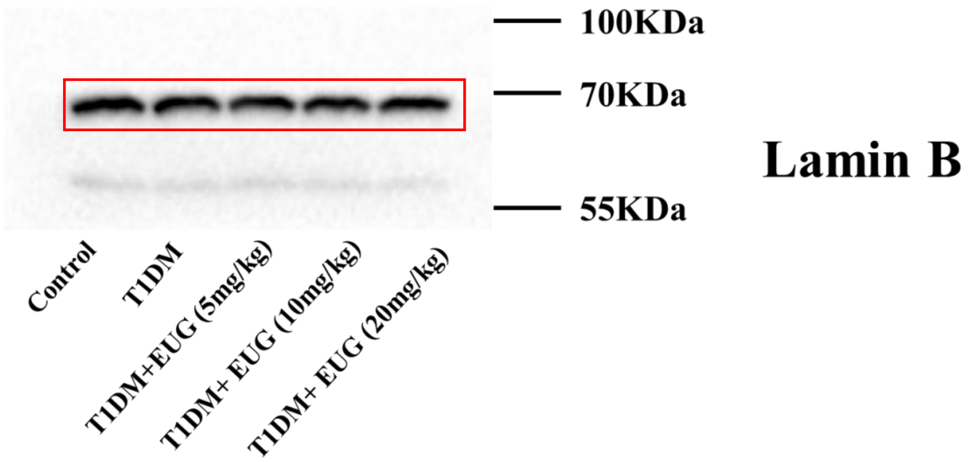
100KDa
70KDa
55KDa
Lamin B
Control
T1DM
T1DM+EUG (5mg/kg)
T1DM+ EUG (10mg/kg)
T1DM+ EUG (20mg/kg)

Full unedited gel for Figure 3D. The red box shows the image used in the manuscript.

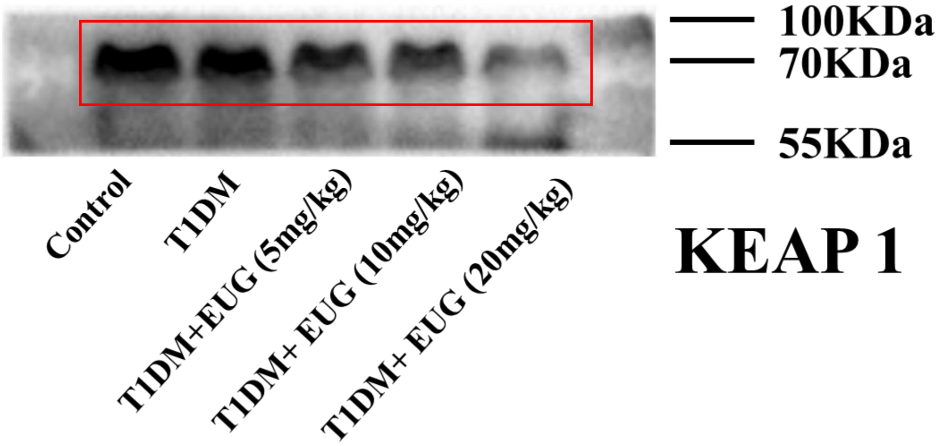

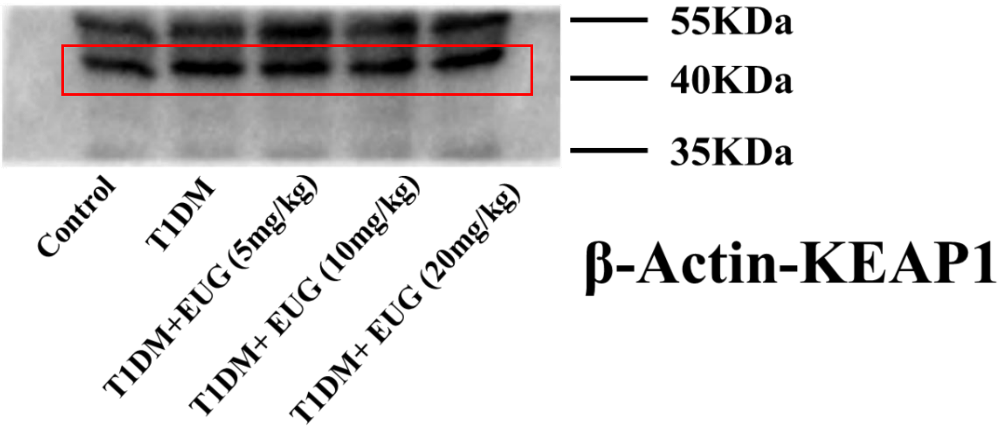

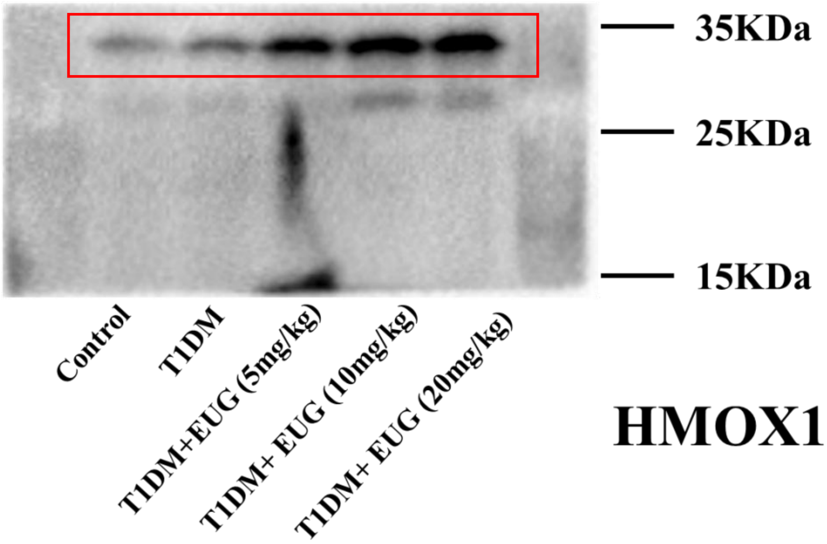

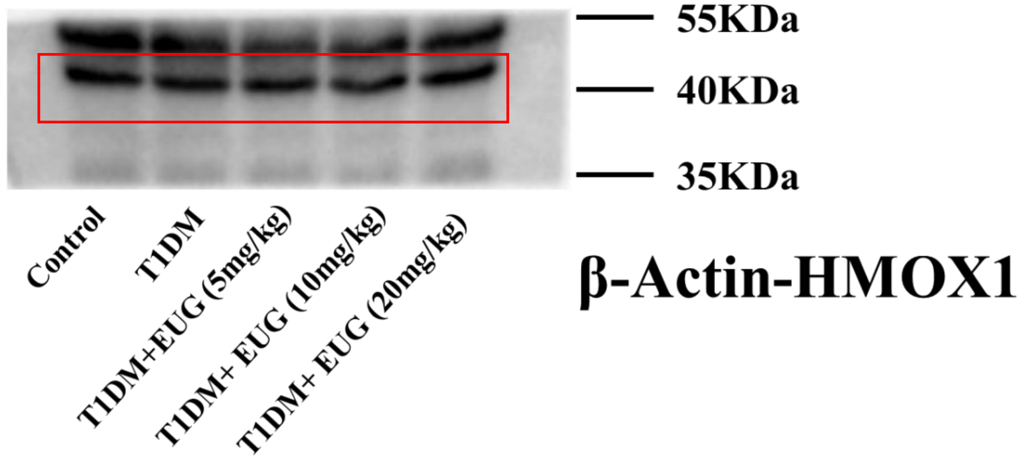
55KDa
40KDa
35KDa
Control
T1DM
T1DM+EUG (5mg/kg)
T1DM+ EUG (10mg/kg)
T1DM+ EUG (20mg/kg)
β-Actin-HMOX1

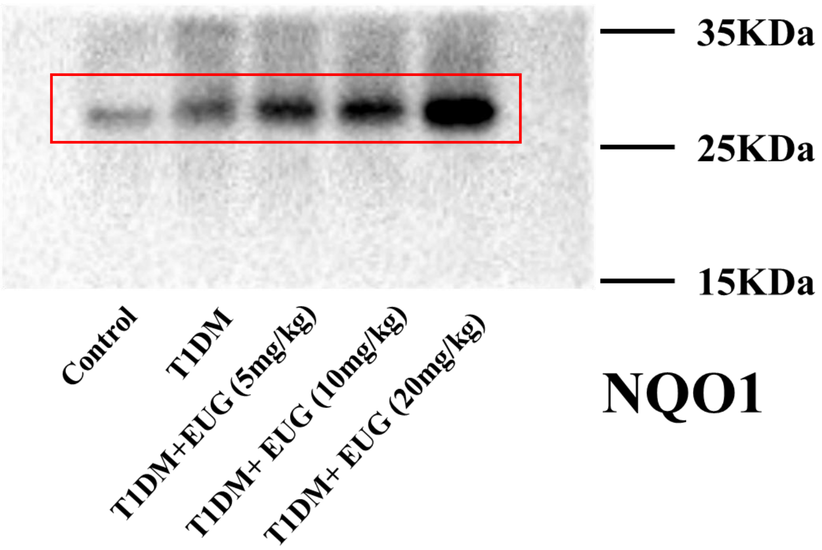
35KDa
25KDa
15KDa
Control
T1DM
T1DM+EUG (5mg/kg)
T1DM+ EUG (10mg/kg)
T1DM+ EUG (20mg/kg)
NQO1

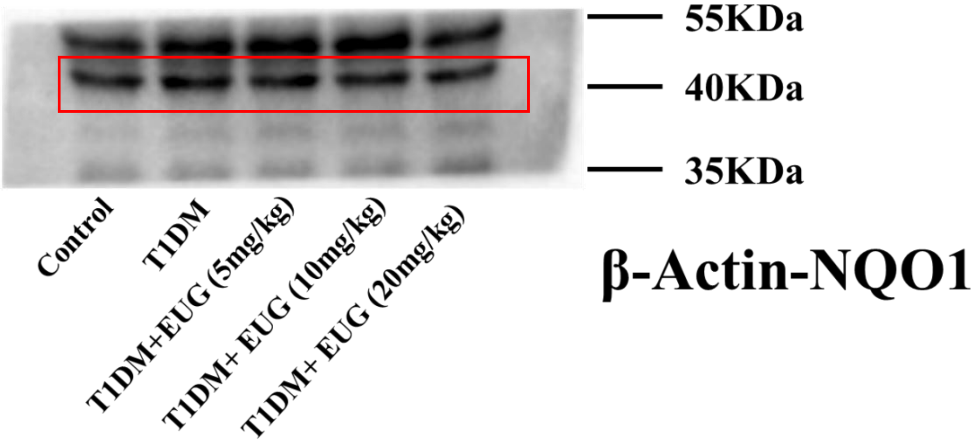
55KDa
40KDa
35KDa
Control
T1DM
T1DM+EUG (5mg/kg)
T1DM+ EUG (10mg/kg)
T1DM+ EUG (20mg/kg)
β-Actin-NQO1
