# Supplementary material for: The protective roles of eugenol on type 1 diabetes mellitus through NRF2-mediated oxidative stress pathway
Source: eLife. 2025 Jan 10;13:RP96600. doi: 10.7554/eLife.96600 (PMC11723580; doi:10.7554/eLife.96600)
Supplement: Figure 6—source data 1. [file elife-96600-fig6-data1.pdf]

Full unedited gel for Figure 6B. The red box shows the image used in the manuscript.

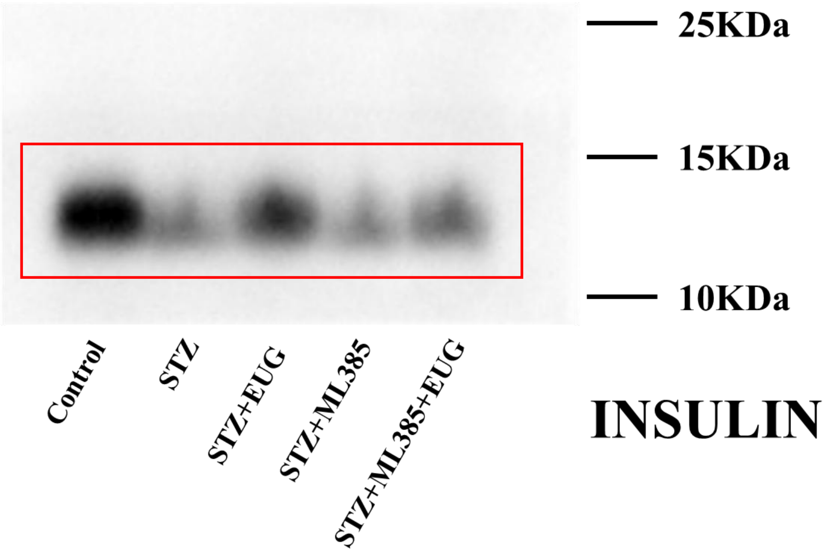

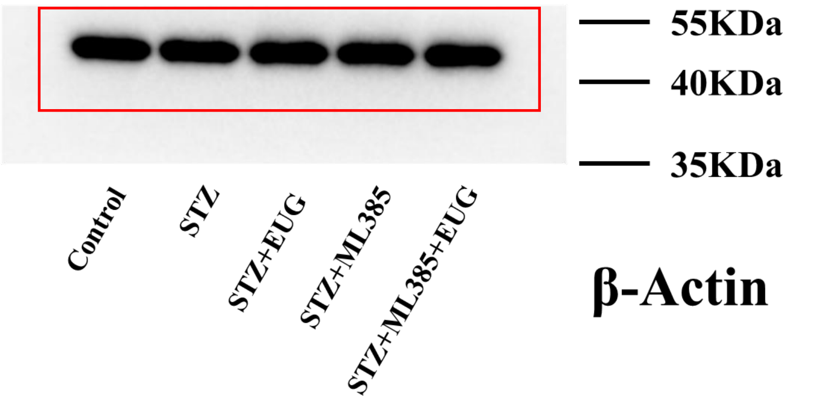

Full unedited gel for Figure 6F. The red box shows the image used in the manuscript.

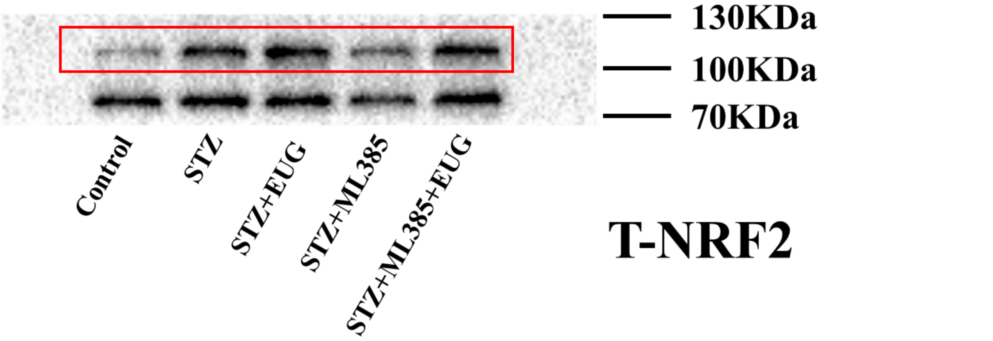

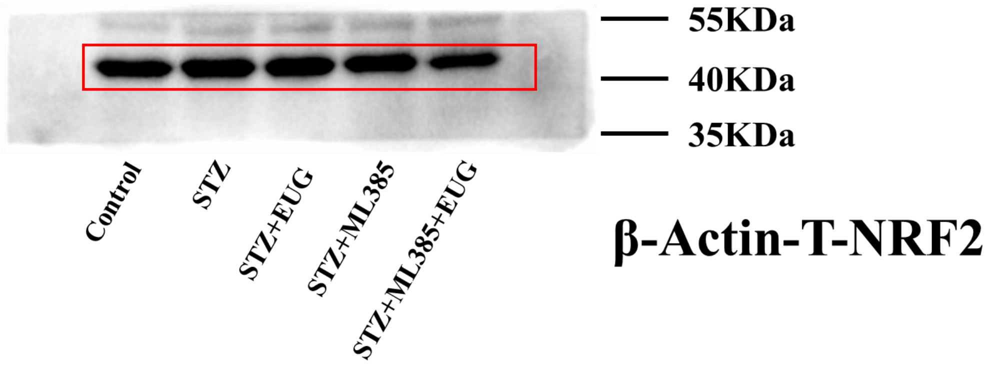

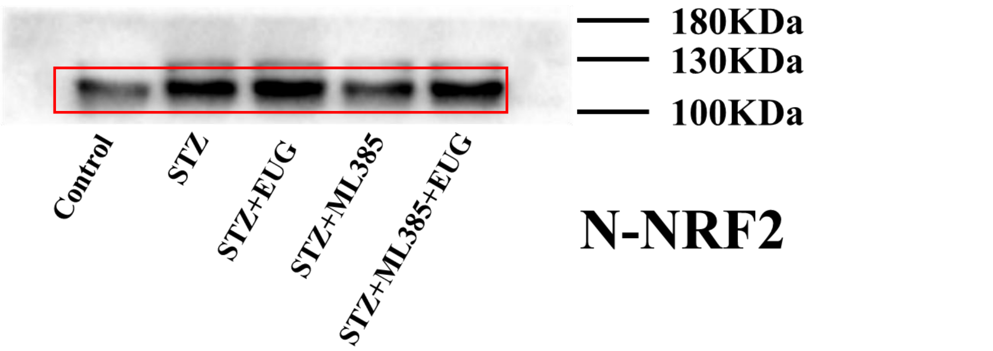

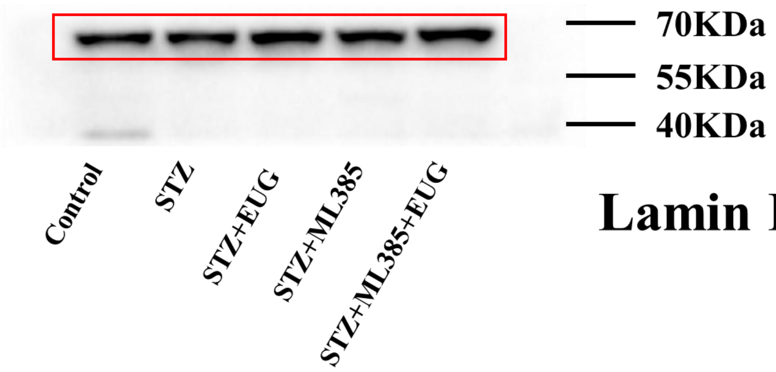

Lamin B-N-NRF2
