# Supplementary material for: The protective roles of eugenol on type 1 diabetes mellitus through NRF2-mediated oxidative stress pathway
Source: eLife. 2025 Jan 10;13:RP96600. doi: 10.7554/eLife.96600 (PMC11723580; doi:10.7554/eLife.96600)
Supplement: Figure 9—source data 1. [file elife-96600-fig9-data1.pdf]

Full unedited gel for Figure 9A. The red box shows the image used in the manuscript.

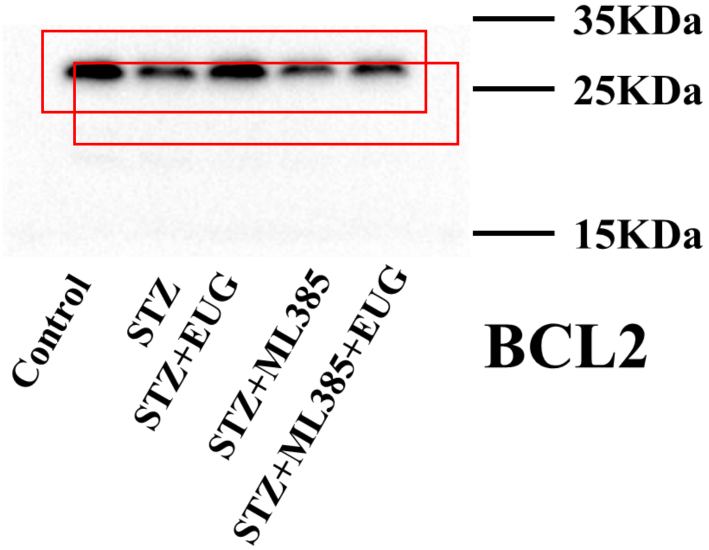

BCL2

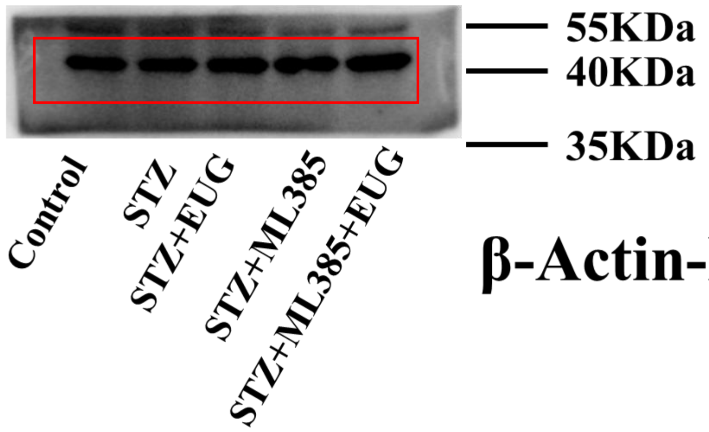

β-Actin-BCL2

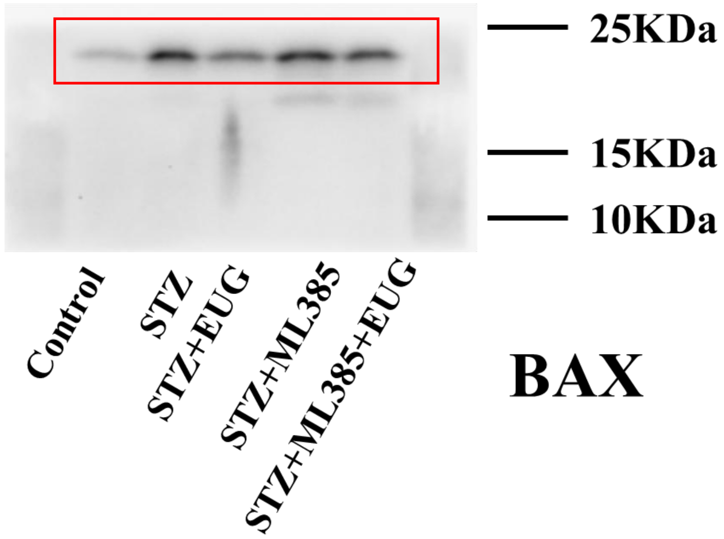

BAX

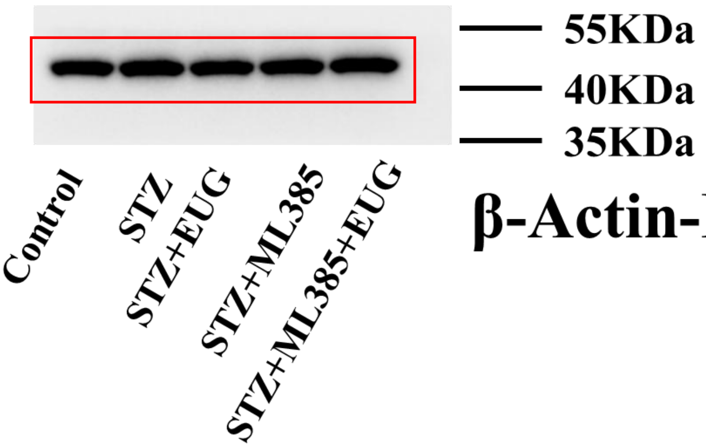

β-Actin-BAX

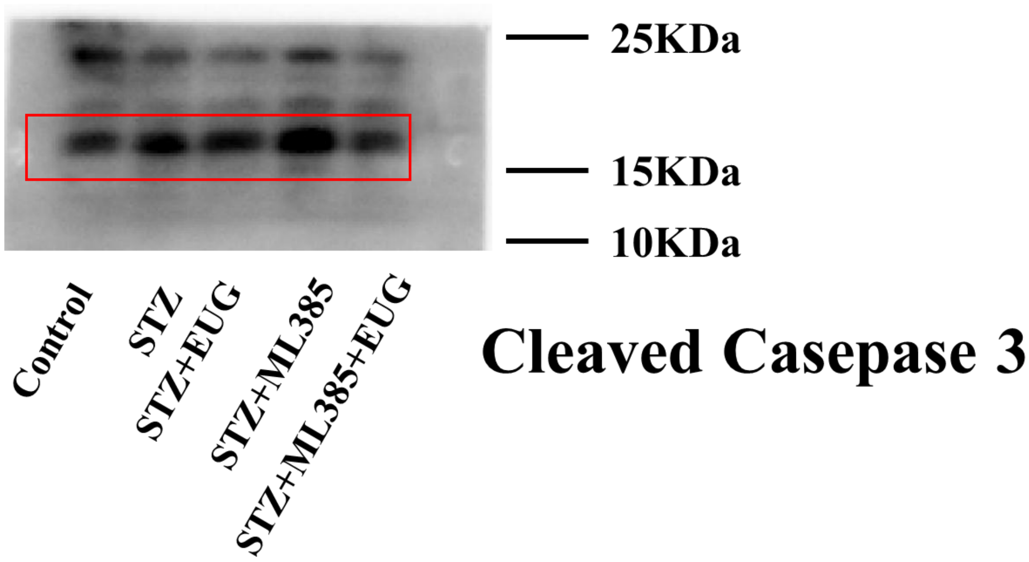

Cleaved Casepase 3

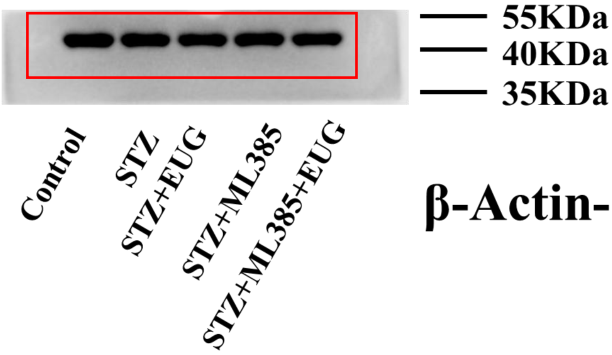

β-Actin-Cleaved Casepase 3
