# Supplementary material for: The protective roles of eugenol on type 1 diabetes mellitus through NRF2-mediated oxidative stress pathway
Source: eLife. 2025 Jan 10;13:RP96600. doi: 10.7554/eLife.96600 (PMC11723580; doi:10.7554/eLife.96600)
Supplement: Supplementary file 2. [file elife-96600-supp2.docx]

**Supplementary File 2. Primer information for mouse**

| **Gene name** | **Primer direction** | **Sequences (5’to 3’)** |
| --- | --- | --- |
| *Ins1* | Forward | CAAACCCACCCAGGCTTTTG |
|  | Reverse | AACGCCAAGGTCTGAAGGTC |
| *Bax* | Forward | ACACTGGACTTCCTCCGTGA |
|  | Reverse | AGAGGAGGCCTTCCCAGC |
| *Bcl2* | Forward | TGAACTGGGGGAGGATTGTG |
|  | Reverse | CAGAGACAGCCAGGAGAAATCA |
| *Nrf2* | Forward | CAGCCATGACTGATTTAAGCAG |
|  | Reverse | CAGCTGCTTGTTTTCGGTATTA |
| *Hmox1* | Forward | TCCTTGTACCATATCTACACGG |
|  | Reverse | GAGACGCTTTACATAGTGCTGT |
| *β-actin* | Forward | CTACCTCATGAAGATCCTGACC |
|  | Reverse | CACAGCTTCTCTTTGATGTCAC |
| *Keap1* | Forward | GACTGGGTCAAATACGACTGC |
|  | Reverse | GAATATCTGCACCAGGTAGTCC |
| *Nqo-1* | Forward | GAAGACATCATTCAACTACGCC |
|  | Reverse | GAGATGACTCGGAAGGATACTG |
